# Construction and validation of an immune infiltration-related risk model for predicting prognosis and immunotherapy response in low grade glioma

**DOI:** 10.1186/s12885-023-11222-5

**Published:** 2023-08-05

**Authors:** Jinna Li, Qing Guo, Rui Xing

**Affiliations:** 1 Department of Oncology, Shengjing Hospital of China Medical University, Shenyang, 110000 China; 2 Department of Neurosurgery, The First Hospital of China Medical University, Shenyang, 110000 China

**Keywords:** Glioma, Immune cell infiltration, Prognosis, Immune checkpoint inhibitor

## Abstract

**Background:**

Low grade glioma (LGG) is considered a heterogeneous tumor with highly variable survival and limited efficacy of immunotherapy. To identify high-risk subsets and apply immunotherapy effectively in LGG, the status and function of immune infiltration in the glioma microenvironment must be explored.

**Methods:**

Four independent glioma cohorts comprising 1,853 patients were enrolled for bioinformatics analysis. We used ConsensusClusterPlus to cluster patients into four different immune subtypes based on immune infiltration. The immune-infiltration signature (IIS) was constructed by LASSO regression analysis. Somatic mutation and copy number variation (CNV) analyses were performed to explore genomic and transcriptomic traits in the high- and low- risk groups. The correlation between response to programmed cell death 1 (PD-1) blockade and the IIS risk score was confirmed in an in vivo glioma model.

**Results:**

Patients were clustered into four different immune subtypes based on immune infiltration, and the high immune infiltration subtype was associated with worse survival in LGG. The high immune infiltration subtype had stronger inflammatory response, immune response and immune cell chemotaxis. The IIS, consisting of EMP3, IQGAP2, METTL7B, SLC1A6 and TNFRSF11B, could predict LGG malignant progression, which was validated with internal clinical samples. M2 macrophage infiltration positively correlated with the IIS risk score. The high-risk group had significantly more somatic mutations and CNVs. The IIS risk score was related to immunomodulatory molecules and could predict immunotherapy clinical benefit. In vivo, immunotherapy-sensitive glioma model exhibited higher IIS risk score and more infiltration of immune cells, especially M2 macrophages. The IIS risk score was decreased in an immunotherapy-sensitive glioma model after anti-PD1 immunotherapy.

**Conclusion:**

Different immune subtypes of LGG had unique immune cell infiltration characteristics, and the high immune infiltration subtype was associated with immunosuppressive signaling pathways. A novel IIS prognostic model based on immune infiltration status was constructed for immunophenotypic classification, risk stratification, prognostication and immunotherapy response prediction in LGG.

**Supplementary Information:**

The online version contains supplementary material available at 10.1186/s12885-023-11222-5.

## Introduction

Gliomas account for nearly 80% of primary malignant brain tumors [1]. Low-grade gliomas (LGGs), which are classified as World Health Organization (WHO) grade II and III gliomas, are well-differentiated, slowly growing and less aggressive. However, LGGs are characterized by high incidence rate, strong heterogeneity and significant prognostic differences. Most LGGs inevitably develop pathological progression and deterioration, and nearly half of patients die from recurrence or metastasis after surgery [2]. At present, the comprehensive treatments for LGG include surgery, radiotherapy, chemotherapy, electric field therapy and targeted therapy, but the clinical prognosis of glioma patients is still not optimistic. Subsets of patients at high risk for recurrence and death may benefit from further systematic treatment and screening out these patients can improve the prognosis of LGG.

Immunotherapy has dramatically changed the clinical outcome of several types of tumors in the past decade. Immune checkpoint inhibitors (ICIs) are the most mature and widely used immunotherapy, and can kill tumor cells by modulating T-cell activity through a variety of pathways. However, because of its low mutation load and because it relatively rarely infiltrates immune effector cells, glioma has a limited response to ICIs [3]. The efficacy of tumor immunotherapy largely depends on the tumor microenvironment (TME), especially the tumor immune microenvironment. As major components of the TME, infiltrating immune cells play a key role in tumor progression and immunotherapy response. Therefore, understanding the infiltrating is essential to improve the immunotherapy response and develop new immunotherapy strategies. Infiltrating immune cells play diverse roles in glioma biology [4, 5]. Macrophages make up the majority of immune cells within glioma, often comprising up to 30% of the tumor mass [3]. Tumor-associated macrophages (TAMs), like their homeostatic counterparts, exhibit plasticity and can polarize to either a proinflammatory or an immunosuppressive state. Immunosuppressive TAMs dominate the glioma microenvironment, which fosters tumor development, contributes to tumor aggressiveness and recurrence, and impedes the therapeutic effect of various treatment regimens [6]. Glioma-associated macrophages and microglia produce low levels of pro-inflammatory cytokines and lack expression of key molecules involved in T-cell co-stimulation, such as CD86, CD80, and CD40, indicating that they may be negative inducers of T-cell response in glioma [7]. The presence of neutrophils was found to be correlated with higher glioma grade [8]. Moreover, high neutrophil count prior to treatment correlates with positive initial response to the vascular endothelial growth factor A (VEGF-A) antibody bevacizumab, and enhanced neutrophil infiltration into tumor tissue is associated with acquired resistance [9]. The glioma immune microenvironment contains multiple factors that drive T-cell exhaustion and metabolic dysfunction, which is one of the reasons for the poor efficacy of immunotherapy in glioma [10]. To apply immunotherapy efficiently in glioma, it is essential to explore the composition and functional status of immune infiltration in the glioma microenvironment and identify high-risk and immunotherapy sensitive patients.

In this study, we investigated the effect of immune infiltration on prognosis and malignant progression of LGG, as well as related signaling pathways and biological processes. We established an immune infiltration-related risk score and evaluated the value of the immune-infiltration signature (IIS) risk score as a predictive factor of prognosis and progressive malignancy in LGG using data from the Chinese Glioma Genome Atlas (CGGA), The Cancer Genome Atlas (TCGA), and GSE16011 datasets. In addition, we explored the correlation of the IIS risk score with immune populations and immunomodulatory molecules. Finally, we verified the potential of the IIS risk score to predict immunotherapy response in vivo. Taken together, the findings of this study suggest that the IIS risk model characterizing the glioma microenvironment is a potential biomarker for immunophenotypic classification, risk stratification, prognostic assessment and prediction of the immunotherapy response in LGG.

## Materials and methods

### Ethics statement and human specimens (patient cohort)

All animal experiments were conducted in accordance with the China Medical University Animal Care and Use Committee guidelines and was approved by the Ethics Committee of Shengjing Hospital of China Medical University (Approval number: 2022PS949K). The human LGG samples used in this study were collected from the First Hospital of China Medical University (CMU samples, Table S1) and was approved by the Ethics Committee of the First Hospital of China Medical University (KLN202286). The histological diagnoses were confirmed according to the 2016 WHO classification guideline by two neuropathologists. The samples were de-identified before being processed. Informed consent was obtained from each patient.

### Cell culture

Murine GL261 cells were obtained from American Type Culture Collection (Manassas, VA) and cultured according to manufacturer’s instructions. DSB cells were generated from a murine spontaneous glioma model previously reported [11, 12]. DSB cells were cultured in RPMI-1640 medium, containing 10% FBS and 1% penicillin/streptomycin (Gibco) at 37°C with 5% CO2.

### Dataset preparation for gene expression and clinical data

Our study involved information on 1,853 glioma samples from four databases. The RNA sequencing (RNA-seq) and clinical data of the TCGA dataset were extracted from the GlioVis portal (http://recur.bioinfo.cnio.es/) [13], including 622 samples (grade II: 224 cases; grade III: 243 cases; glioblastoma: 155 cases). The RNA-seq and clinical data of the CGGA dataset were downloaded from the CGGA website (http://www.cgga.org.cn/). The CGGA 325 RNA-seq dataset included 310 samples (grade II: 105 cases; grade III: 67 cases; glioblastoma: 138 cases). The CGGA 693 RNA-seq dataset included 657 samples (grade II: 172 cases; grade III: 248 cases; glioblastoma: 237 cases). The GSE16011 dataset was downloaded from Gene Expression Omnibus (GEO) Datasets, including 264 samples (grade II: 24 cases; grade III: 85 cases; glioblastoma: 155 cases).

### Immune infiltration estimation and consensus clustering

To identify infiltrating immune cells in the TME, the supplied cell markers of 22 immune cell types were downloaded from “CIBERSORT”. The infiltration level of each immune cell type in the TME was quantified by a single-sample gene set enrichment analysis (ssGSEA) algorithm using the “GSVA” R package. The value acquired by ssGSEA represented the relative abundance of each infiltrating immune cell type in each sample.

The consensus clustering of 22 immune cell types was performed by the “ConsensusClusterPlus” R package with the following parameters: reps = 1000, pItem = 0.8, and pFeature = 1. The optimal number of clusters was determined by heatmap and delta diagram analyses.

### Functional enrichment analysis

Differentially expressed genes (DEGs) between cluster I and cluster IV were identified using the “limma” R package with the standards of log2 (fold change) > 3 and *p* < 0.0001 in the TCGA and CGGA 325 RNA-seq datasets (Table S2). Gene ontology biological process (GO-BP) and Kyoto Encyclopedia of Genes and Genomes (KEGG) analyses were performed by DAVID (https://david.ncifcrf.gov/tools.jsp) based on these DEGs [14, 15]. Gene set enrichment analysis (GSEA, http://www.broadinstitute.org/gsea/index.jsp) was performed to identify whether the hallmark gene sets showed significant differences between cluster I and cluster IV. Statistical significance was determined by the normalized enrichment score (NES) and false discovery rate (FDR) [16]. Hallmark gene sets with |NES|> 1.5 and FDR < 0.25 were defined as significantly enriched hallmark gene sets. The KEGG signaling pathways of common DEGs from TCGA and CGGA 325 RNA-seq datasets (Table S3) were analyzed by ClueGO of Cytoscape software [17].

### Construction of the immune-infiltration signature (IIS)

DEGs between cluster I and cluster IV were obtained by the ‘limma’ R package in the TCGA and CGGA 325 RNA-seq datasets, respectively. An adjusted p value < 0.0001 and |log2 (fold change) |> 3 were used as the cut-off values (Table S3). A least absolute shrinkage and selection operator (LASSO) Cox penalized regression model was performed using the “glmnet” R package to construct the IIS prognostic model based on the common DEGs of TCGA and CGGA 325 RNA-seq datasets [18]. The IIS risk score was calculated by weighting the Cox regression coefficients, and the formula was as follows: IIS risk score = ΣDEGs gene expression * coefficient.

### Kaplan‒Meier survival analysis and receiver operating characteristic (ROC) curve

The median IIS risk score was employed as the cut-off value to classify patients into the high- and low-risk groups. Kaplan‒Meier survival curves were plotted using the “survival” R package. To verify the accuracy and validity of the IIS risk score, the area under the curve (AUC) values for 1-, 3- and 5-year overall survival were calculated via the “pROC” R package [19, 20].

### Construction and verification of the predictive nomogram

A nomogram was constructed with the “rms” R package in the TCGA and CGGA 325 RNA-seq datasets [21]. To construct the nomogram, univariate Cox regression analysis of clinical data and IIS risk score was performed. Age, WHO grade, 1p19q status, IDH status and IIS risk score had *p* < 0.05 in the univariate analysis, and they were integrated into the predictive nomogram. A calibration curve was used to assess whether the predicted value of the model was consistent with the probability of the outcome [22]. The C-index indicated the probability that the predicted outcomes were consistent with the actual observed outcomes. Age, WHO grade, 1p19q status, IDH status, IIS risk score and nomogram score were compared through C-index analysis.

### Microenvironment composition parameters and cell population analyses

Tumor purity, immune score and stromal score were calculated according to the method described previously [8]. xCell analysis was performed at https://xcell.ucsf.edu/ [23]. In addition, the TIMER, quanTIseq and EPIC methods were performed via TIMER2.0 (http://timer.cistrome.org/) [24].

### Tracking Tumor Immunophenotype (TIP) analysis

TIP analysis was calculated by http://biocc.hrbmu.edu.cn/TIP/ [25], which was used to evaluate the stepwise events of the cancer-immunity cycle in the anticancer immune response.

### Somatic mutation and copy number variation (CNV) analysis

CNV profile and somatic mutation data were collected from the TCGA RNA-seq dataset (https://portal.gdc.cancer.gov/). GISTIC 2.0 was used to calculate CNVs associated with the IIS risk score. GISTIC values < -1 or > 1 were defined as gene deletions or gene amplifications. The “maftools” R package was used to visually analyze the somatic mutation data.

### RNA isolation and reverse-transcription quantitative PCR (RT‒qPCR)

Total RNA was isolated from GL261 and DSB mouse tissue samples using TRIzol reagent (Invitrogen), according to the manufacturer’s instructions. Total RNA was reverse-transcribed into cDNA with Prime-Script RT Master Mix (TaKaRa). RT‒qPCR was performed in a thermal cycler (Roche) with SYBR Green Master Mix (TaKaRa). The following conditions were used: 1 cycle of 95 °C for 30 s, followed by 40 cycles of a two-step cycling program (95 °C for 5 s; 60 °C for 30 s). The mRNA expression of target genes was calculated by the 2-ΔΔCt method and normalized to *Gapdh* mRNA expression. The PCR primer sequences are listed in Table S4.

### Intracranial mouse model

Four-to-six-week-old male C57BL/6 mice purchased from Beijing Vital River Laboratory Animal Technology were used to establish an intracranial glioma murine model. A total of 5 × 10^5^ GL261 or DSB glioma cells transduced with control lentiviral firefly luciferase vectors were intracranially (i.c.) injected into mice to create GL261 and DSB-bearing murine models. Programmed cell death 1 (PD-1) antibody (BioCell, No. CP151) was intraperitoneally (i.p.) injected at a dose of 10 μg/g body weight after glioma cells were implanted for 6, 9 and 12 days. All mice were sacrificed by cervical dislocation under anesthesia with 2% isoflurane inhalation when exhibiting neurological signs, or more than 20% of body weight loss.

Tumor tissues were harvested at day 26 and subjected to RT‒qPCR, H&E and immunohistochemistry (IHC) analyses. The antibody panel and qPCR primer sequences are listed in Table S3. For the detection of TAMs source and polarization status and T-cell status in GL261 and DSB mouse samples, RT-qPCR and IHC were performed to detect the expression of M1 macrophage and M2 macrophage polarization markers and CD3/CD4/CD8 T-cell markers.

### Statistical analysis

Statistical analysis was performed primarily with GraphPad Prism 7 software. One-way ANOVA and two-tailed t tests were used to calculate the significant quantitative differences between and among groups. Kaplan‒Meier survival analysis was performed using R (version 3.6.0). The log-rank test was used to evaluate the difference between the stratified groups. Univariate and multivariate Cox regression analyses were used to estimate the prognostic value of the IIS risk score. A *p* value < 0.05 was defined to indicate statistical significance.

## Results

### Correlation between immune infiltration and clinical prognosis in LGG

**Fig. 1:**
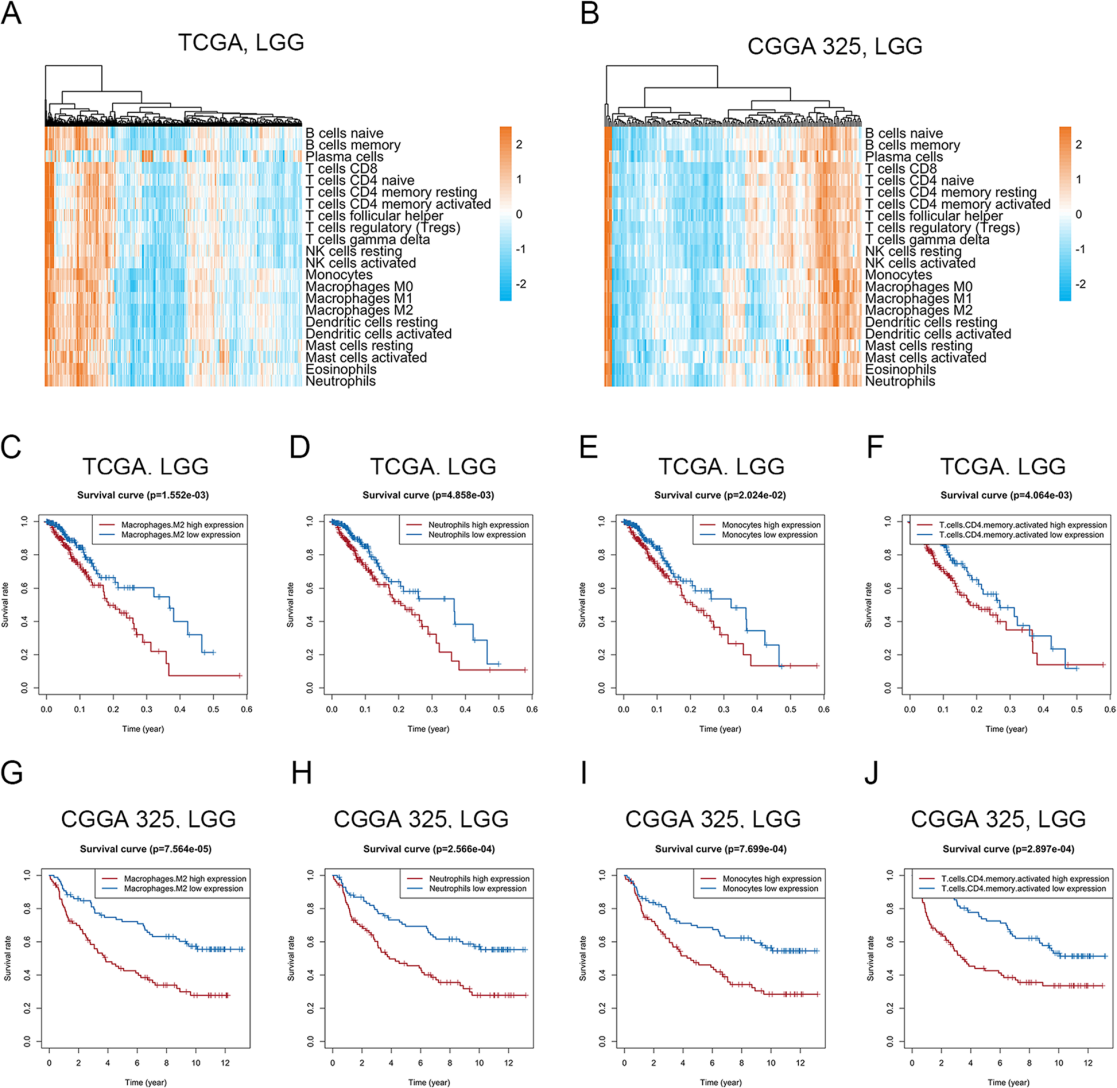
Correlation between immune infiltration and clinical prognosis in LGG. **A-B**. Heatmap showed the heterogeneity of immune cell infiltration in LGG in the TCGA (**A**) and CGGA 325 (**B**) RNA-seq datasets. **C-J**. Kaplan–Meier diagram showed the correlation between infiltration of some immune cells and OS in the TCGA (**C-F**) and CGGA 325 (**G-J**) RNA-seq datasets

Differences in immune cell infiltration were observed in LGG (Fig. 1A-B and Fig. S1A-B). Higher infiltration of M2 macrophages, neutrophils, monocytes and CD4 memory-activated T-cells was associated with worse survival in the TCGA, CGGA 325 and CGGA 693 RNA-seq datasets (Fig. 1C-J and Fig. S1C-G), although neutrophils, monocytes and CD4 memory-activated T-cells did not show differences in survival in the GSE16011 microarray dataset (Fig. S1H-J). These results indicate that the status of immune cell infiltration in LGG was heterogeneous, and infiltration of some immune cells was correlated with unfavorable prognosis.

### Four immune subtypes based on immune cell infiltration in LGG

**Fig. 2:**
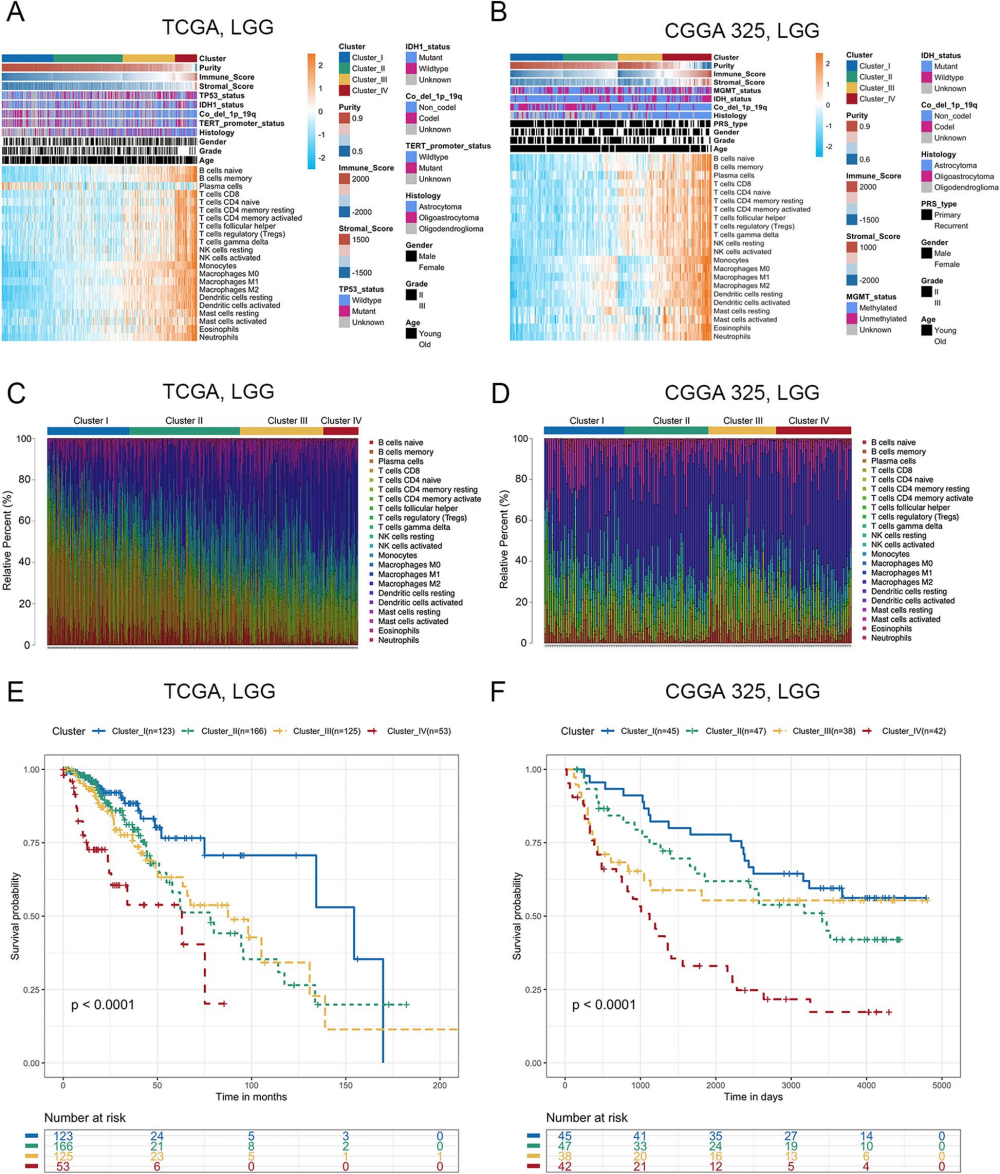
Four immune subtypes with different immune cell infiltration status in LGG. **A-B**. Heatmap showed the immune cell infiltration status of four immune subtypes of LGG in the TCGA (**A**, cluster I: *n* = 123; cluster II: *n* = 250; cluster III: *n* = 166; cluster IV: *n* = 53) and CGGA 325 (**B**, cluster I: *n* = 45; cluster II: *n* = 38; cluster III: *n* = 43; cluster IV: *n* = 47) RNA-seq datasets. **C-D**. Barplot showed the distribution of 22 immune cell types in the TCGA (**C**) and CGGA 325 (**D**) RNA-seq datasets. **E–F**. Kaplan–Meier survival curve showed different overall survival in cluster I, II, III and IV in the TCGA (**E**) and CGGA 325 (**F**) RNA-seq datasets

Unsupervised clustering analysis was carried out by the “ConsensusClusterPlus” R package. The samples reconstructed from the TCGA, CGGA 325, CGGA 693 and GSE16011 datasets were grouped into four clusters, and heatmaps showed differences in the expression of 22 immune cell populations between these four clusters (Fig. 2A-B and Fig. S2A-B). In cluster I, the level of immune cell infiltration was low, defined as the ‘low immune infiltration subtype’, while in cluster IV, the level of immune cell infiltration was high, defined as the ‘high immune infiltration subtype’. The immune score and stromal score increased from cluster I to IV, while tumor purity gradually decreased from cluster I to IV (Fig. 2A-B and Fig. S2A-B). We evaluated the heterogeneity of immune infiltration in LGG by analyzing the proportion of different immune cell types and found that as the tumor progressed from cluster I to IV, the proportion of macrophages showed an increasing trend (Fig. 2C-D and Fig. S2C-D). Survival analysis showed that there were significant differences among the four clusters, and cluster IV had the worst survival time (Fig. 2E-F and Fig. S2E-F). Taken together, immune cell infiltration status was unique in different immune subtypes and could be regarded as a prognostic marker in LGG.

### The high immune infiltration subtype was associated with immune-related signaling pathways

**Fig. 3:**
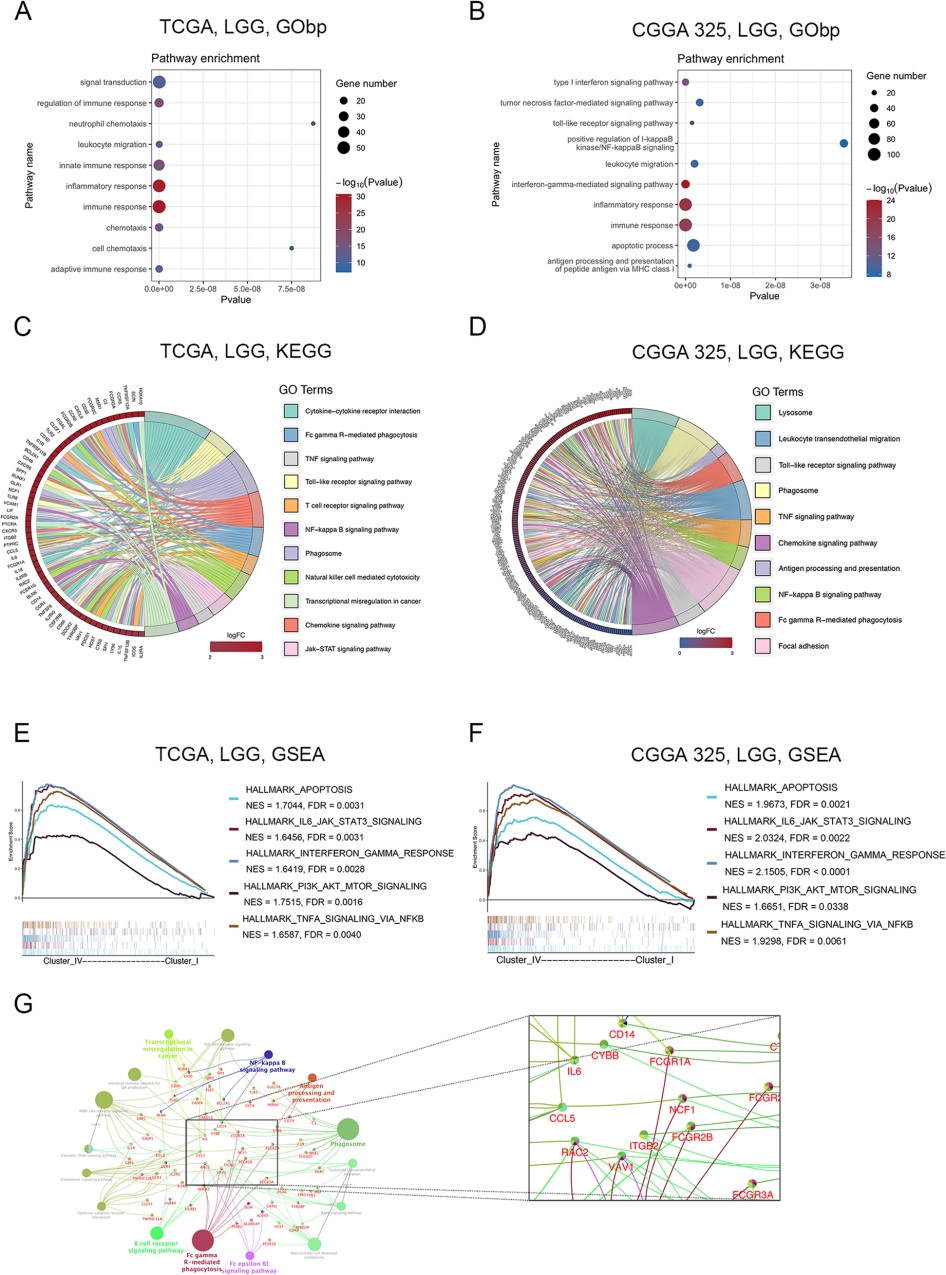
Signal pathways and biological processes enriched in the high immune infiltration subtype. **A-B**. GO enrichment analysis of the high immune infiltration subtype in the TCGA (**A**) and CGGA 325 (**B**) RNA-seq datasets. **C-D**. KEGG RNA-seq pathway analysis of the high immune infiltration subtype in the TCGA RNA-seq dataset (**C**) and CGGA 325 RNA-seq dataset (**D**). **E–F**. GSEA hallmark analysis of the high immune infiltration subtype in the TCGA (**E**) and CGGA 325 (**F**) RNA-seq datasets. **G**. KEGG pathway analysis with overlapping DEGs between cluster I and cluster IV in the TCGA and CGGA 325 RNA-seq datasets (adjust *p* < 0.0001, |logFC|> 3)

We performed GO and KEGG enrichment analyses based on the DEGs (*p* < 0.0001, log FC > 3) between cluster I and cluster IV. GO analysis showed that the DEGs of the high immune infiltration subtype were mainly involved in immune response, inflammatory response, and leukocyte migration (Fig. 3A-B). KEGG analysis indicated that related pathways of the high immune infiltration subtype were mainly enriched in immune-related signaling pathways, including phagosome, chemokine signaling pathway, Toll-like receptor signaling pathway, Fcγ-R-mediated phagocytosis and TNF signaling pathway, as well as caner-associated signaling pathways, such as the NF-κ-B signaling pathway and Jak-STAT signaling pathway (Fig. 3C-D). Furthermore, we performed GSEA based on hallmark gene sets and found that the high immune infiltration subtype was mainly enriched in apoptosis, IL6-JAK-STAT3, interferon-γ response, PI3K-AKT-MTOR and TNFA-NFκB signaling pathway (Fig. 3E, F). We selected DEGs (adjusted *p* < 0.0001, |logFC|> 3) between cluster I and cluster IV and took the common genes from TCGA and CGGA 325 RNA-seq datasets. Pathway analysis by Cytoscape software revealed that the high immune infiltration subtype was mainly related to phagosomes, Fcγ R-mediated phagocytosis, antigen processing and presentation, and NF-κ-B signaling pathway (Fig. 3G). The key genes participating in these signal transduction pathways included IL-6, CCL5, RAC2, ITGB5, FCGR2A, CYBB, CD14 and VAV1 (Fig. 3G). However, GO and KEGG analyses revealed that the DEGs of the low immune infiltration subtype were mainly enriched in biological processes such as GABAergic synapse and glutamatergic synapse (Fig. S3A-B). Collectively, these results indicate that the high immune infiltration subtype was related to a higher number of activated immune-related signaling pathways and promoted the formation of a tumor-promoting immune microenvironment in LGG.

### The IIS risk score was constructed based on immune cell infiltration status and was a predictive factor for progressive malignancy in LGG

**Fig. 4:**
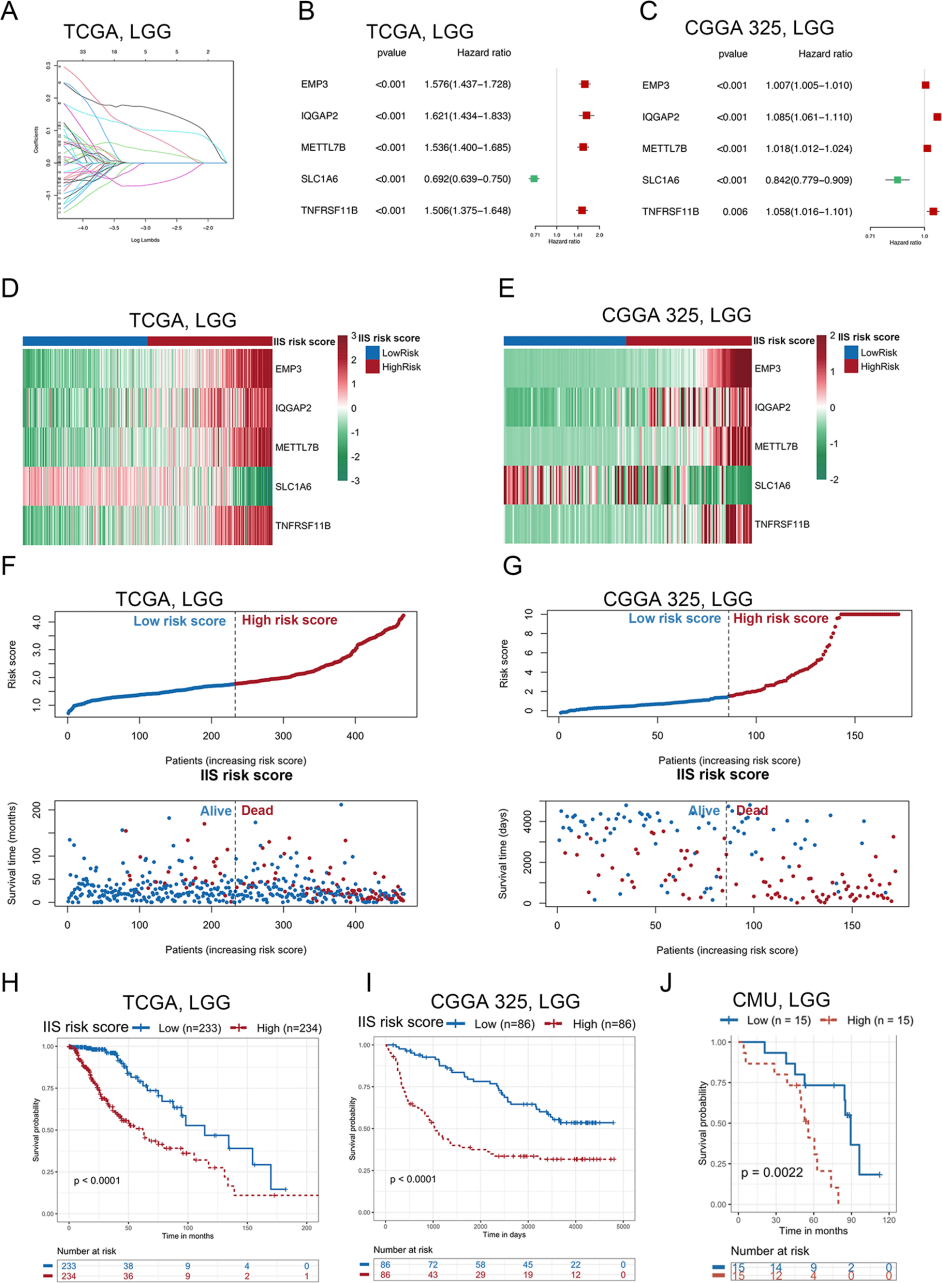
Construction and validation of IIS in LGG. **A**. Least absolute shrinkage and selection operator (LASSO) regression was performed to calculate the minimum criteria and coefficient in TCGA RNA-seq dataset. **B-C**. Univariate Cox regression analysis of five IIS genes included in the LASSO risk model in the TCGA (**B**) and CGGA 325 (**C**) RNA-seq datasets. **D-E**. Correlation of five IIS genes expression with risk score in the TCGA (**D**) and CGGA 325 (**E**) RNA-seq datasets. **F-G**. Distribution of IIS risk score and survival status in the TCGA (**F**) and CGGA 325 (**G**) RNA-seq datasets. H-I. Kaplan–Meier survival curve based on the IIS risk score in the TCGA (**H**) and CGGA 325 (I) RNA-seq datasets (log-rank *p*-value < 0.0001). **J**. Kaplan–Meier survival curve based on the IIS risk score in CMU glioma tissues (log-rank *p*-value = 0.0022)

The DEGs between cluster I and cluster IV were defined by the “limma” R package (adjusted *P* < 0.0001 and |logFC|> 3.0). Then, we selected the common DEGs (*n* = 233) from TCGA and CGGA 325 RNA-seq datasets (Fig. S4A) and performed LASSO Cox regression analysis (Fig. 4A and Fig. S4B). We identified five potential risk-related genes in the prognostic signature. Among these five genes, SLC1A6 was defined as a protective gene with a hazard ratio (HR) of < 1, whereas EMP3, IQGAP2, METTL7B and TNFRSF11B were defined as risk-conferring genes with HRs of > 1 (Fig. 4B-C and Fig. S4C-D). The expression levels of these five genes were used to predict the risk level in LGG. The formula was as follows: IIS risk score = (0.1722 * EMP3 expression) + (0.0910 * IQGAP2 expression) + (0.0390 * METTL7B expression) + (-0.0687 * SLC1A6 expression) + (0.1017 * TNFRSF11B expression). We applied the risk formula and stratified patients based on the median IIS risk score. The heatmap revealed the mRNA expression patterns of these five genes in the high- and low-risk groups (Fig. 4D-E and Fig. S4E-F). The risk curve and scatterplot showed that patients with high-risk scores had a relatively high risk of mortality (Fig. 4F-G and Fig. S4G-H). Kaplan‒Meier survival analysis showed that the high-risk group had worse survival than the low-risk group (Fig. 4H-I and Fig. S4I-J), which was verified in CMU glioma tissues (Fig. 4J). Univariate and multivariate COX regression analysis revealed the IIS risk score was an independent prognostic factor in LGG (Table 1).

**Table 1:** Univariate and multivariate Cox regression analyses of clinical information and IIS risk score in the TCGA RNAseq dataset

Variable	Univariate Regression			Multivariate Regression		
	HR	95% CI	*p* Value	HR	95% CI	*p* Value
**Age**	1.0565	1.0403–1.0730	< 0.0001	1.0523	1.0311–1.074	< 0.0001
**Grade**	2.9543	1.9359–4.5084	< 0.0001	2.0654	1.1244–3.794	0.0194
**IDH1_status**	0.2190	0.1336–0.3590	< 0.0001	0.6732	0.3077–1.473	0.3218
**Co_del_1p_19q**	0.4679	0.2557–0.8564	0.0138	0.6901	0.3488–1.365	0.2866
**IIS risk score**	3.7881	2.9773–4.8197	< 0.0001	2.2626	1.4059–3.641	0.0008

Moreover, we established a nomogram to better validate the predictive accuracy and translational potential (Fig. S5A-B). The C-index of the nomogram was 0.8693 in the TCGA RNA-seq dataset and 0.7972 in the CGGA 325 RNA-seq dataset, which was significantly higher than other constituent factors (IIS risk score: 0.8206 (TCGA) and 0.7223 (CGGA 325); age: 0.7937 (TCGA) and 0.5600 (CGGA 325); WHO grade: 0.6568 (TCGA) and 0.6829 (CGGA 325); 1p19q status: 0.5712 (TCGA) and 0.6690 (CGGA 325), IDH status: 0.7183 (TCGA) and 0.6039 (CGGA 325)) (Fig. S5C-D). The calibration plot showed a high degree of consistency between the predicted probability and actual 1-, 3- and 5-year survival rates (Fig. S5E-F). To estimate the validity of the nomogram for predicting survival, ROC curves were generated based on the 1-, 3- and 5-year survival rates, and the respective AUC values were 90.7%, 92.0% and 83.8% in the TCGA dataset (Fig. S5G). We validated these results in the CGGA seq 325 dataset, and the 1-, 3- and 5-year AUC values were 82.3%, 87.9% and 89.6%, respectively (Fig. S5H).

**Fig. 5:**
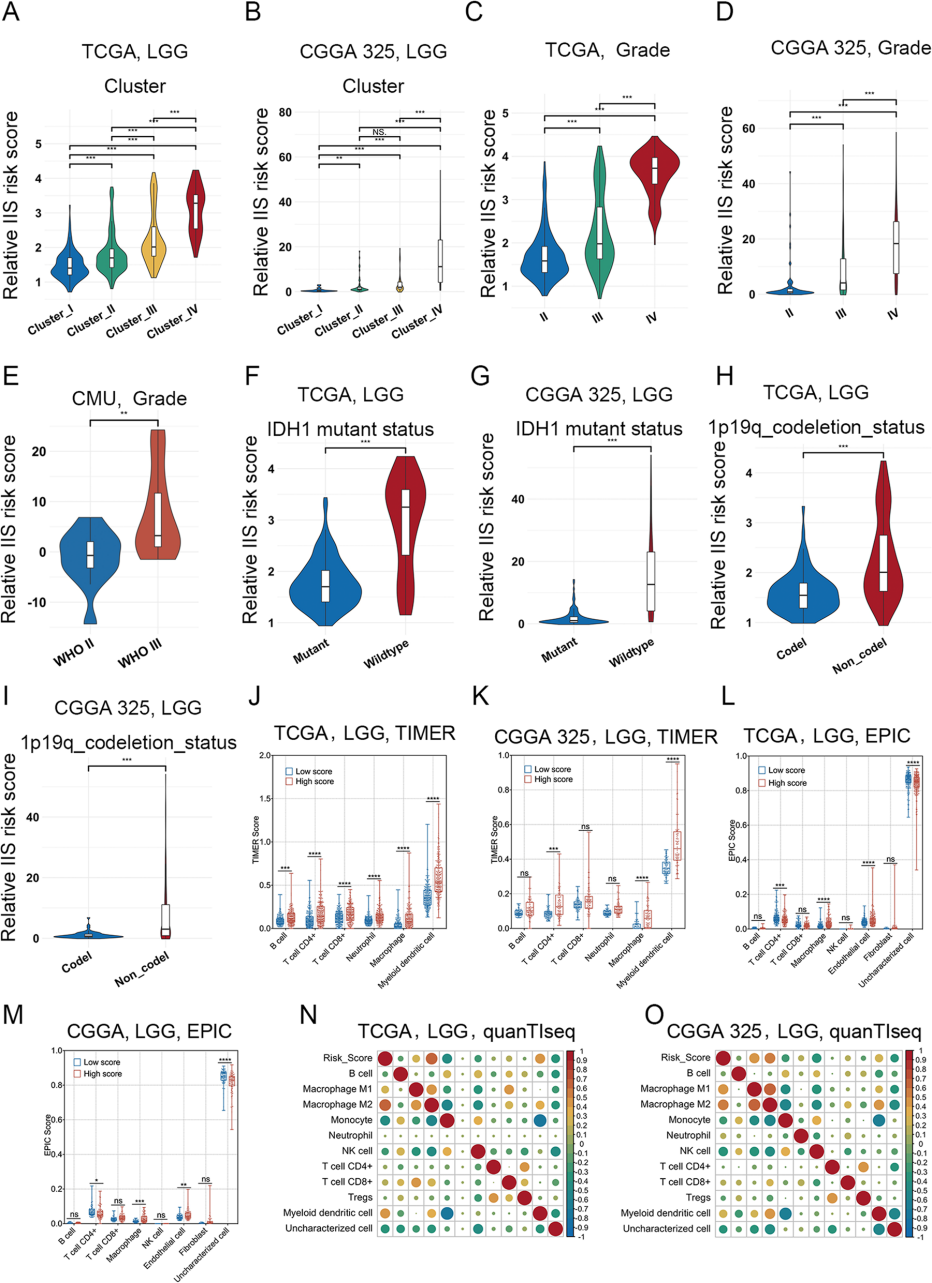
Correlation of IIS risk score with clinicopathological features and non-tumor cell populations in the TME. **A-B**. IIS risk score varied among four clusters in the TCGA (**A**) and CGGA 325 (**B**) RNA-seq datasets. **C-E**. Violin plots revealed that patients with different WHO grades had different IIS risk scores in the TCGA, CGGA 325 RNA-seq datasets and CMU glioma tissues. **F-I**. Violin plots revealed that patients with different clinicopathological features (including IDH1 mutation status and 1p/19q co-deletion status) had different IIS risk scores in the TCGA and CGGA 325 RNA-seq datasets. **J-K**. Immune cell infiltration of high- and low-risk group through TIMER in the TCGA (**J**) and CGGA 325 (**K**) RNA-seq datasets. **L-M**. Immune cell infiltration of high- and low-risk group through EPIC in the TCGA (**L**) and CGGA 325 (**M**) RNA-seq datasets. **N–O**. Correlation matrix of all 11 immune cell populations with IIS risk score through quanTIseq in the TCGA (**N**) and CGGA 325 (**O**) RNA-seq datasets. *p < 0.05; **p < 0.01; ***p < 0.001; ****p < 0.0001; NS, no significant

There were significant differences in the IIS risk score among the four clusters, and the IIS risk score increased gradually from cluster I to cluster IV (Fig. 5A-B and Fig. S6A-B). The IIS risk score was correlated with some clinicopathological features in LGG. Patients with high WHO grade, wild-type IDH and 1p19q non codeletion showed higher IIS risk scores (Fig. 5C-I and Fig. S6C-G). Patients with MGMT methylation had a lower IIS risk score than those with unmethylated MGMT (Fig. S6H). Glioblastoma patients had a higher IIS risk score than astrocytoma, oligoastrocytoma and oligodendroglioma patients (Fig. S6I-L). Taken together, the IIS risk score was a predictive factor for prognosis and progressive malignancy in LGG.

### Correlation of the IIS risk score with non-tumor cell populations in the TME

Glioma purity and related nontumor components in the TME confer important clinical, genomic and biological implications. Correlation analysis was performed between the IIS risk score and microenvironment landscape of LGG. TIMER immune cell infiltration evaluation revealed that patients with a high IIS risk score had more infiltration of CD4 + T-cells, macrophages, and myeloid dendritic cells (Fig. 5J-K). EPIC analysis of nontumor cell infiltration showed that patients with a high IIS risk score had more infiltration of macrophages and endothelial cells, and less infiltration of CD4 + T cells (Fig. 5L-M). QuanTIseq analysis showed that the infiltration of M2 macrophages had the most significant positive correlation with the IIS risk score (R ≥ 0.3, *p* < 0.05) (Fig. 5N-O and Fig. S7A-B). We analyzed the correlation of the IIS risk score with the stromal score, immune score and tumor purity in LGG with the ESTIMATE package and found that the IIS risk score was positively correlated with the stromal score and immune score, but negatively correlated with tumor purity (Fig. S7C-D). Further cell population enrichment analysis by xCell also revealed that the infiltration of M2 macrophages had a positive relevance to the IIS risk score (Fig. S7C-D), indicating that macrophages, specifically M2 macrophages, were important nontumor cells in regulating the immune microenvironment in LGG.

### LGG with different IIS risk scores had distinct genomic and transcriptomic spectra

To reveal the molecular characteristics associated with the expression pattern of the IIS risk score, we collected available somatic mutation and CNV information in the TCGA dataset. The high-risk group had higher mutation frequencies, and its top ten mutant genes were slightly different from those of the low-risk group. Moreover, the mutation rates of common mutant genes in glioma in the high-risk group were significantly higher than those in the low-risk group, such as IDH1 (70% vs. 50%), TP53 (42% vs. 36%), ATRX (33% vs. 25%) and EGFR (8% vs. 5%) (Fig. S8A-B). The high-risk group also had significantly more copy number deletion events than the low-risk group (Fig. S8C-D). The region with the most frequent genomic deletion in the high-risk group was 9p21.3, which contained interferon alpha and interferon epsilon, including IFNA1, IFNA2, IFNA4, IFNA5, IFNA6, IFNA7, IFNA8, IFNA10, IFNA13, IFNA14, IFNA16, IFNA17 and IFNE (Fig. S8C-D, *p* = 5.97E-05). On the other hand, the region with the most frequent genomic amplification in the high-risk group was 7p11.2, which contained EGFR (Fig. S8C-D, *p* = 2.53E-12). These results suggested that LGG patients with high IIS risk scores had more significant somatic mutation frequencies and CNVs.

### The IIS risk score was associated with immunomodulatory molecules and could predict the response to immunotherapy

The balance of costimulatory receptors and coinhibitory receptors plays a critical role in the regulation of the immune microenvironment and has been well studied in autoimmune and cancer therapy [26]. There were significant differences in immune costimulatory molecules and immune coinhibitory molecules in the LGG samples with different risk scores (Fig. S9A-B). There were six immunomodulatory factors with Pearson *r* > 0.5 and *p* < 0.05, including two costimulatory immune molecules, TNFSF12A and TNFRSF14, and four immune co-inhibitory molecules, PDCD1LG2, LGALS3, LAIR1 and CD276, which had a positive correlation with the IIS risk score (Fig. S9C-D).

To explore whether the IIS risk score could predict immunotherapy response, we applied the IIS risk score to the Van_allen dataset, the sequencing data from metastatic melanoma treatment with cytotoxic T lymphocyte-associated antigen-4 (CTLA-4) blockade (ipilimumab). The response group showed a higher IIS risk score than the nonresponse group (Fig. S9E). Patients receiving ipilimumab exhibited a significant survival benefit in the high-risk group (*p* = 0.0311, Fig. S9F). The prognostic accuracy of the IIS risk score was confirmed by ROC curve analysis, with an AUC value of 0.7329 (*p* = 0.0188, Fig. S9G). These data indicated that the IIS risk score was associated with some immunomodulatory molecules and could serve as a potential predictor of immunotherapy response.

### The IIS risk score in the immunotherapy-sensitive glioma model was higher and decreased after anti-PD1 immunotherapy in vivo

**Fig. 6:**
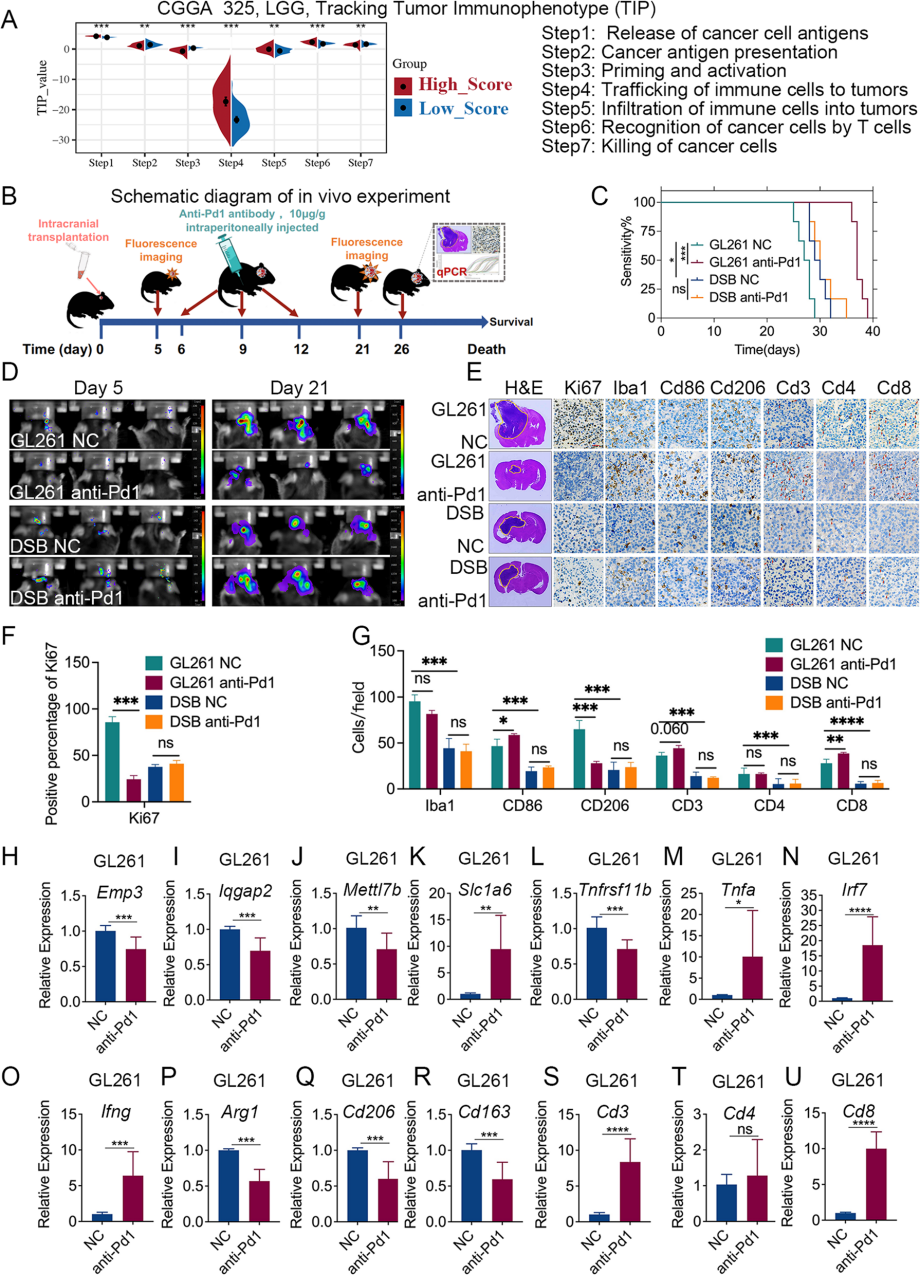
The IIS risk score could predict the degree of immune cell infiltration and the response to immunotherapy in glioma. **A**. IIS risk score was associated with each stepwise events of the Cancer-Immunity Cycle according to TIP analysis in the CGGA 325 RNA-seq dataset. **B**. Schematic diagram of the experimental process with anti-PD1 immunotherapy in glioma model mice. **C**. Survival plot of mice in indicated groups (*n* = 6, log-rank test). **D**. Representative bioluminescence brain images at indicated time points after intracranial transplantation of GL261 or DSB glioma cells (*n* = 6). **E**. Representative H&E (left) and immunohistochemical staining (right) of Ki67, Iba1, Cd86, Cd206, Cd3, Cd4 and Cd8 in mice intracranial tumor from indicated groups. (*n* = 3, Scale bar, 20 μm). **F**-**G**: IHC analysis of Ki67, IBA1, CD86, CD206, CD3, CD4, and CD8 in tumors from GL261 and DSB-bearing C57BL/6 mice under different treatments (One way ANOVA, *n* = 3). **H**-**U**. qPCR analyses of indicated markers in tumor tissue from C57BL/6 mice intracranially transplanted with GL261 glioma cells (*n* = 3, t-test). *p < 0.05; **p < 0.01; ***p < 0.001; ****p < 0.0001; ns, no significant

The correlation between the IIS risk score and the seven steps of the anticancer immune response was further analyzed by TIP. A high IIS risk score was significantly associated with a lower degree of cancer antigen presentation (Step 2), priming and activation (Step 3) and killing of cancer cells (Step 7) but was related to a higher degree of release of cancer cell antigens (Step 1), trafficking of immune cells to tumors (Step 4), infiltration of immune cells into tumors (Step 5) and recognition of cancer cells by T-cells (Step 6) (Fig. 6A). To further verify the results of the bioinformatics analysis, we constructed GL261 and DSB glioma murine models in vivo. Anti-PD1 immunotherapy was used in these two glioma models (Fig. 6B). We found that in GL261 mice, PD-1 blockade significantly prolonged the survival period (Fig. 6C) and inhibited glioma growth (Fig. 6D-G). In DSB mice, PD-1 blockade failed to prolong the survival period and did not inhibit glioma growth (Fig. 6C-G). In the GL261 glioma model, the IIS risk score was significantly decreased after anti-PD1 immunotherapy (the expression levels of Emp3, Iqgap2, Mettl7b, Tnfrsf11b significantly decreased, and expression of Slc1a6 significantly increased) (Fig. 6H-L), while in the DSB glioma model, there was no significant change in the IIS risk score before and after anti-PD1 immunotherapy (Fig. S11A-E).

In the GL261 glioma model, which was defined as an immunotherapy-sensitive glioma model, increased expression of M1 macrophage markers (*Tnfa, Irf7*, and *Ifng*) and decreased expression of M2 macrophage markers (*Arg1, Cd206* and *Cd163*) were found after treatment with anti-PD1 immunotherapy (Fig. 6E, 6G and 6M-R). In addition, CD3 + T lymphocytes, especially CD8 + T lymphocytes were significantly increased after anti-PD1 immunotherapy, and there was no significant change in CD4 + T lymphocytes (Fig. 6E, 6G and 6S-U). In contrast, there was less immune cell infiltration and a low IIS risk score in the DSB glioma model. In the DSB glioma model, the immune cell population and IIS risk score did not change significantly after anti-PD1 immunotherapy, and the response to PD-1 blockade was negligible (Fig. 6E, 6G and S10A-N).

In the control group, the IIS risk score of the GL261 murine model was significantly higher than that of the DSB murine model (higher expression of Emp3, Iqgap2, Mettl7b and Tnfrsf11b and lower expression of Slc1a6 in the GL261 murine model) (Fig. S11A-E). The GL261 murine model with high immune cell infiltration in the control group had significantly worse survival than the DSB murine model (Fig. 6C). H&E staining and Ki67 immunohistochemical staining showed that the tumor size and proliferation ability of the GL261 murine model without anti-PD1 immunotherapy were significantly higher than those of the DSB murine model without anti-PD1 immunotherapy (Fig. 6E-F). We speculated that differences in survival and tumor size may be due to different types and numbers of infiltrating immune cells in tumor tissue. We further explored the correlation between immune cell infiltration and the response to anti-PD1 immunotherapy by IHC and qPCR analysis. IHC analysis showed that the expression levels of Iba1 (marker of macrophages), CD206 (marker of M2 macrophages), CD3 (marker of T lymphocytes) and CD8 (marker of CD8 + T lymphocytes) in the GL261 murine model were higher than that in the DSB murine model in the control group, which indicated that the infiltration of macrophages, especially M2 macrophages, and T lymphocytes, especially CD8 + T lymphocytes, were significantly higher in the GL261 murine model than in the DSB murine model (Fig. 6E and 6G). Similar conclusions were obtained from the qPCR results (Fig. S11F-N). The above results indicated that the glioma murine model with a high IIS risk score had more immune cell infiltration, especially of M2 macrophages, and was more sensitive to immune checkpoint blockade therapy.

## Discussion

ICIs have recently demonstrated remarkable clinical benefits in various solid tumors and have attracted great attention. However, because of the “cold phenotype” of glioma and drug resistance caused by multiple factors, immunotherapy is still limited in the clinical application of glioma [27]. Therefore, understanding the unique immune status and TME of glioma is necessary for the effective application of immunotherapy. There are various peripheral immune components in the glioma microenvironment, including CD8 cytotoxic T lymphocytes (CTLs), CD4 helper T (Th) cells, macrophages, myeloid-suppressor cells (MDSCs), natural killer (NK) cells, neutrophils and Treg cells. Compared to other tumors, their infiltration rates in glioma are significantly lower [27]. Considerable evidence suggests that the diversity and density of infiltrating immune cells in the TME affect the modulation of the immune response, the efficacy of immunotherapy and the prognosis of tumors [28]. Tumor infiltrating lymphocytes (TILs) are the most important components of the immune response for most solid tumors. Compared to other tumor types, TILs are less abundant in central nervous system tumors [27]. TAMs play a critical role in tumor angiogenesis, neoplasia, metastasis and immune escape [29, 30]. Massive TAM infiltration is closely associated with poor prognosis [31]. Contrary to their proinflammatory function during infection, neutrophils are conducive to tumor progression and metastasis. Tumor associated neutrophils (TANs) promote tumor deterioration by mediating angiogenesis in the glioma microenvironment [32]. This study comprehensively analyzed immune infiltration status, immune microenvironment characteristics, and prognostic characteristics of different immune subtypes of LGG, as well as their different functions. We found that immune cell infiltration can effectively predict the prognosis and malignant phenotype of LGG patients. Consistent with previous studies, M2 macrophages, neutrophils and monocytes were negatively correlated with survival in LGG. The LGG samples were clustered into four immune subtypes based on the degree of immune cell infiltration, and the proportion of macrophages in the samples showed an increasing trend from the low immune infiltration subtype to the high immune infiltration subtype. Although cluster IV had the most robust immune infiltration, the complex immunosuppressive microenvironment in samples in this cluster, marked by high infiltration of macrophages and low infiltration of CD8 + CTLs, may result in the worst survival among the four immunotypes. Due to the combined negative regulation of macrophages, neutrophils, monocytes, and T lymphocytes, the overall microenvironment of LGG is immunosuppressive.

We constructed a robust immune infiltration-related signature integrating the expression levels of EMP3, IQGAP2, METTL7B, SLC1A6 and TNFRSF11B to predict the prognosis of LGG patients. Some of these genes have been previously reported to be linked to tumor progression and tumor immunity. EMP3 is involved in various biological processes, including cell proliferation, apoptosis, adhesion and migration [33]. EMP3 is regarded as an immunosuppressive factor in glioblastoma because it mediates M2 TAM infiltration and suppresses T-cell infiltration to facilitate tumor progression [34]. IQGAP2 has been reported as a tumor suppressor in hepatocellular cancer, prostate cancer, and gastric cancer [35]. However, IQGAP2 is a negative prognostic factor and is associated with immunosuppression in diffuse large B-cell lymphoma [36]. IQGAP2 was defined as a risk-conferring gene in this study and its role in LGG needs further study. Previous studies have reported the role and function of METTL7B in glioma which modulates tumor proliferation, migration, invasion, epithelial–mesenchymal transition and the immune response [37, 38]. In glioma, high expression level of METTL7B indicates poor prognosis and an immunosuppressive microenvironment [39, 40]. TNFRSF11B is a prognostic factor in colon cancer and suppresses memory CD4 + T cell infiltration in the colon cancer microenvironment [41]. In this study, we constructed an IIS prognostic model based on immune infiltration and validated its predictive accuracy and translational potential in LGG. However, further experiments are needed to demonstrate the specific mechanisms by which these five genes are involved in immune cell infiltration and the formation of an immunosuppressive microenvironment in LGG.

Mutations in the isocitrate dehydrogenase genes (IDH1 and IDH2) define a unique glioma subtype associated with an immunosuppressive tumor microenvironment [42]. Although gliomas with IDH mutation are less proliferative and have better survival outcomes, they secrete more extracellular vesicles that can lead to immune evasion and are more immunosuppressive than those released from IDH-wild gliomas [42–44]. IDH-mutant gliomas exhibit reduced NK and CD8 + T-cell infiltration compared to IDH-wild gliomas and are resistant to innate cytotoxic immune mechanisms, indicating they are inherently capable of escaping immune surveillance [45]. IDH1 mutation caused downregulation of leukocyte chemotaxis, resulting in reduced infiltration of immune cells and suppression of the tumor-associated immune system [45]. In this study, a high IIS risk score represented high infiltration of immune cells and was associated with poor prognosis. IDH1-mutant gliomas showed relatively lower IIS risk scores than IDH1-wild gliomas, which is consistent with the favorable prognosis of IDH1 mutant gliomas with reduced immune infiltration [45].

Compared with other tissues, the brain TME has a distinct composition, which is mainly composed of functionally diverse astrocytes and pro-tumorigenic macrophages, but exclusive of infiltrating lymphocytes [46]. Previous studies have shown that M2 macrophage infiltration is negatively correlated with survival in several solid tumors. TAMs inhibit tumor proliferation through the proinflammatory "M1" phenotype in the early stage of glioma, while TAMs are dominated by the "M2" phenotype in advanced glioma, which usually induces an immunosuppressive response and tumor immune escape [27]. In this study, the high immune infiltration subtype (cluster IV) had the highest proportion of macrophages and the worst prognosis. Immune cell population analysis showed that M2 macrophages had the highest correlation with the IIS risk score. The above results are consistent with the role of TAMs in forming an immunosuppressive glioma microenvironment and promoting tumor progression. Although the high IIS risk score and the high immune infiltration subtype had more immune cell infiltration, the immune microenvironment was mainly dominated by macrophages, monocytes, neutrophils and other immune cells that lead to immune escape or immunosuppression. This may explain why glioma with high immune infiltration status may have an immunosuppressive microenvironment and poor prognosis. In this study, the infiltration of macrophages, especially M2 macrophages, showed a positive correlation with the IIS risk score. Given that the immune microenvironment of glioma is dominated by macrophages, monocytes and neutrophils, the high immune infiltration status of LGG patients with a high IIS risk score may contribute to an immunosuppressive microenvironment and poor prognosis.

Analysis of clinical sequencing data after immunotherapy revealed that patients with high IIS risk scores had better response to immune checkpoint inhibitor treatment. In vivo experiments found that the immunotherapy-sensitive glioma murine model had a higher IIS risk score and more immune cell infiltration. The immunotherapy-unresponsive glioma murine model DSB had a low IIS risk score and low degree of immune cell infiltration, similar to the “immune desert type”. These results further indicated that the IIS risk score reflected the degree and type of immune cell infiltration in glioma. Although the infiltrating immune cells in the GL261 mice were mainly M2 macrophages, which may reflect an immunosuppressive microenvironment, there was still a certain degree of infiltration of T lymphocytes, including CD8 + T cells, which may have caused the GL261 mice to have some response to immunotherapy.

TAMs express high levels of PD-1, and PD-1 expression increases with tumor progression [47]. PD-1 + TAMs are mainly M2 macrophages [47]. PD-1 expression by TAMs inhibits phagocytosis and tumor immunity [47]. Blocking PD-1/PD-L1 pathway in TAMs can play an antitumor role by increasing the phagocytosis of macrophages on tumors [47]. In addition, anti-PD-1/PD-L1 immunotherapy can reverse depleted CD8 + T-cells and promote CD8 + T-cells to secrete interferon-γ (IFN-γ) [48]. IFN-γ can inhibit the migration of macrophages and promote the transformation of M2 macrophages into M1 macrophages [49]. Neoadjuvant immunotherapy can activate exhausted tumor-infiltrating T cells, improve their TCR activity and metabolic activity, and enable CD4 + regulatory T cells to turn into effector cells [50]. Our study demonstrated that the IIS risk score could predict the response to anti-PD1 immunotherapy in glioma. In the immunotherapy-sensitive glioma murine model, M2 macrophages decreased, M1 macrophages increased, CD8 + T-cells increased, the IIS risk score decreased, and the survival was improved after treatment with PD-1 blockade. As mentioned in previous studies, PD-1 inhibitors overcome the restriction of T-cell activity induced by PD-1[51, 52], and this study also showed an increase in T lymphocytes after anti-PD1 treatment. The increased T lymphocytes may have reversed the immunosuppressive microenvironment, resulting in a corresponding decrease in M2 macrophages. The reduction in M2 macrophages may be related to changes in the immune microenvironment after immunotherapy and the direct effects of PD-1 inhibitors on M2 macrophages. On the one hand, anti-PD1 immunotherapy can reverse the role of depleted CD8 + T-cells and play a role in killing tumor cells. On the other hand, anti-PD1 immunotherapy may inhibit the PD-1/PD-L1 signaling pathway of M2 macrophages and promote the transformation of M2 macrophages into M1 macrophages. As mentioned earlier, the IIS risk score was found to be significantly correlated with M2 macrophages. Macrophages are the main immune cell subpopulations in the glioma microenvironment [46]. The proportion of M2 macrophages decreased after immunotherapy, leading to a reversal of the immunosuppressive microenvironment, which may explain why the IIS decreased after immunotherapy in the immunotherapy-sensitive group.

Some immunomodulatory factors have been reported to be related to escape of the antitumor immune response and to mediate resistance to ICIs by modulating immune cell function. In this study, immune checkpoint molecules such as LAIR1 and PDCD1LG2 were significantly positively correlated with the IIS risk score, implying that they are involved in the regulation of immune infiltration in LGG. LAIR1, an immune inhibitory receptor expressed on the majority of immune cell subsets, delivers an inhibitory signal after binding to collagen-like domains and confers poor prognosis in several cancer types [53]. LAIR1 signaling results in the loss of immune function in the TME, and suppression of T-cell, NK cell, monocyte, and dendritic cell activation and function [54]. PD-1 binding to PDCD1LG2 (PD-L2) inhibits the immune activation of T-cells and negatively regulates the immune response [55, 56]. Conversely, PD-L2 binging to repulsive guidance molecule b (RGMb) can activate the function of T cells [56]. The correlation between PD-L2 expression and prognosis is variable, which may be due to the unique immunosuppressive effect of PD-L2 in different malignant tumors [55]. These immune checkpoint molecules might be novel targets to overcome the immunosuppressive microenvironment of immune-excluded tumors [56].

## Conclusions

This study comprehensively elucidates the composition and functional status of immune infiltration in the glioma microenvironment and identifies different immunophenotypes with unique characteristics of the immune microenvironment. We constructed and validated a novel IIS prognostic model based on immune infiltration status for immunophenotypic classification, risk stratification, prognostic assessment and potential efficacy prediction of immunotherapy in LGG, which may help to identify high-risk glioma cases early and promote more individualized immunotherapy to obtain better clinical outcomes. Therapies targeting M2 macrophages or key genes in the IIS prognostic model combined with ICIs might become therapeutic strategies.

### Supplementary Information

**Additional file 1: Fig. S1.** Correlation between immune infiltration and clinical prognosis in LGG.

**Additional file 2: Fig. S2.** Four immune subtypes with different immune cell infiltration status in LGG.

**Additional file 3: Fig. S3.** Signal pathways and biological processes enriched in the low immune infiltration subtype.

**Additional file 4: Fig. S4.** Construction and validation of IIS in LGG.

**Additional file 5: Fig. S5.** Establishment and validation of IIS risk score nomogram.

**Additional file 6: Fig. S6.** Correlation between IIS risk score and clinicopathological features.

**Additional file 7: Fig. S7.** Correlation of IIS risk score with non-tumor immune populations in the TME.

**Additional file 8: Fig. S8.** LGG with different IIS risk score had distinct genomic and transcriptomic spectrum.

**Additional file 9: Fig. S9.** IIS risk score was associated with immunomodulatory molecules and could predict response of immunotherapy.

**Additional file 10: Fig. S10.** There were no significant changes in IIS risk score and immune cell infiltration in the immunotherapy unresponsive group.

**Additional file 11: Fig. S11.** The IIS risk score could predict the degree of immune cell infiltration and the response to immunotherapy in glioma.

**Additional file 12: Table S1.** Clinicopathological information of CMU glioma samples.

**Additional file 13: Table S2.** The DEGs between Cluster I and Cluster IV in the TCGA and CGGA 325 RNA-seq datasets.

**Additional file 14: Table S3.** Overlapping DEGs between Cluster I and Cluster IV in the TCGA and CGGA 325 RNA-seq datasets.

**Additional file 15: Table S4.** The RT-qPCR primer sequences and antibodies were used in this article.

## Data Availability

The datasets supporting the conclusions of this article are available in the TCGA dataset in https://portal.gdc.cancer.gov/, CGGA (Chinese Glioma Genome Atlas) dataset in http://www.cgga.org.cn/, GEO dataset in https://www.ncbi.nlm.nih.gov/gds/, DAVID 6.8 in https://david.ncifcrf.gov/tools.jsp, GSEA in https://www.gsea-msigdb.org/gsea/index.jsp and GSVA in http://www.bioconductor.org.

## Abbreviations

LGG: Low grade glioma
IIS: Immune-infiltration signature
CNV: Copy number variation
PD-1: Programmed cell death 1
WHO: World Health Organization
ICIs: Immune checkpoint inhibitors
TME: Tumor environment
VEGF-A: Vascular endothelial growth factor A
IFN-γ: Interferon-γ
PD-L1: Programmed cell death ligand-1
IDH: Isocitrate dehydrogenase
TAMs: Tumor associated macrophages
Treg: Regulatory T
CGGA: Chinese Glioma Genome: Atlas
TCGA: The Cancer Genome Atlas
RNA sequence: RNA-seq
GEO: Gene Expression Omnibus
ssGSEA: Single-sample gene set enrichment analysis
DEGs: Differentially expressed genes
GO-BP: Gene-ontology biological-processes
KEGG: Kyoto Encyclopedia of Genes and Genomes
GSEA: Gene Set Enrichment Analysis
NES: Normalized enrichment score
FDR: False discovery rate
LASSO: Least absolute shrinkage and selection operator
ROC: Receiver operating characteristic
AUC: Area under the curve
TIP: Tracking tumor immunophenotype
RT-qPCR: Reverse-transcription quantitative PCR
IHC: Immunohistochemistry
CTLA-4: Cytotoxic T lymphocyte-associated antigen-4
CTLs: Cytotoxic T lymphocytes
Th: Helper T
MDSCs: Myeloid-suppressor cells
NK: Natural killer
TILs: Tumor infiltrating lymphocytes
TANs: Tumor associated neutrophils
PD-L2: PDCD1LG2
RGMb: Repulsive guidance molecule b
