# Supplementary figures and images for: Construction and validation of an immune infiltration-related risk model for predicting prognosis and immunotherapy response in low grade glioma
Source: BMC Cancer. 2023 Aug 5;23:727. doi: 10.1186/s12885-023-11222-5 (PMC10403952; doi:10.1186/s12885-023-11222-5)

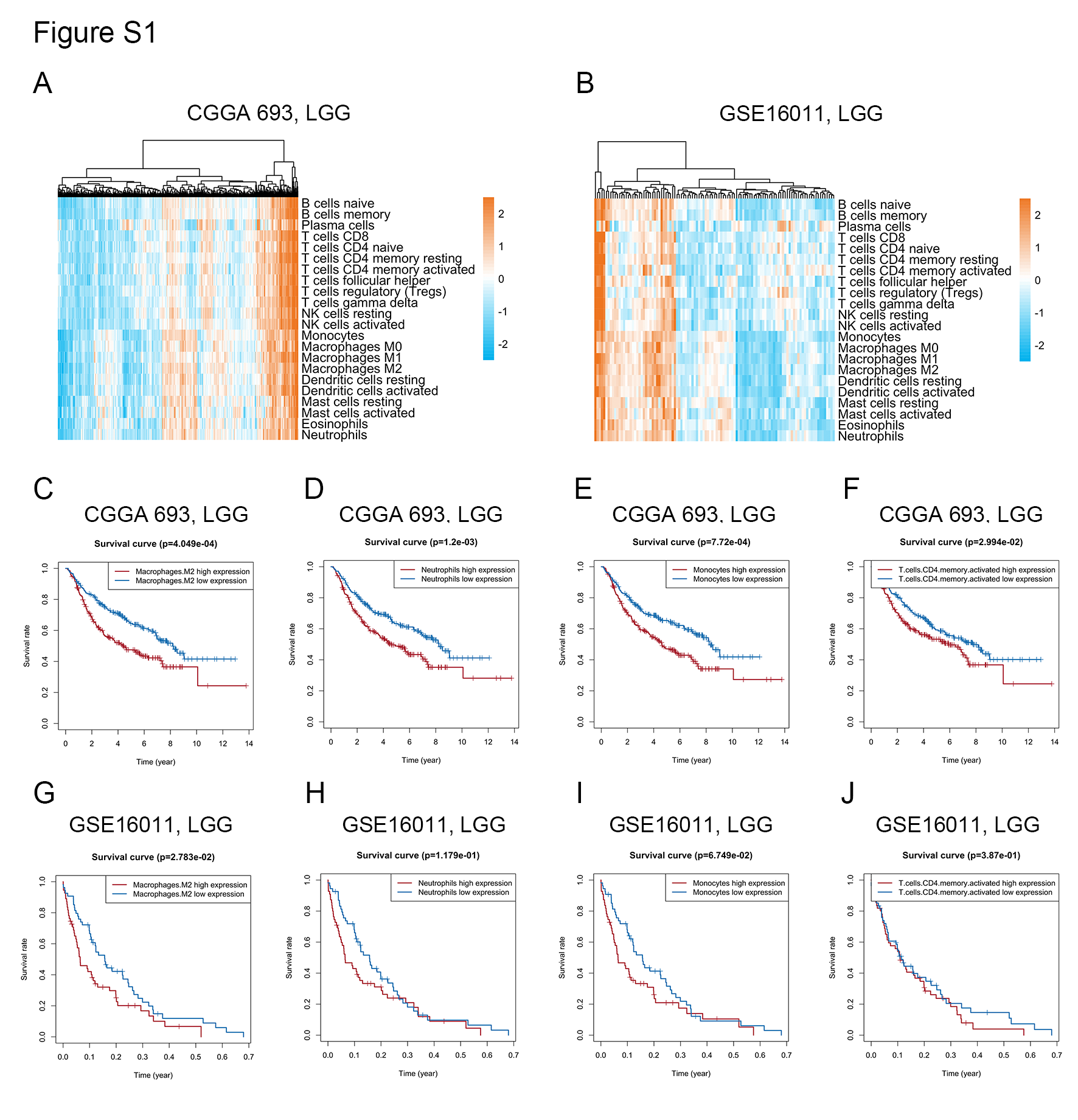

Supplement: Supplementary file 1 — Additional file 1: Fig. S1. Correlation between immune infiltration and clinical prognosis in LGG. [file 12885_2023_11222_MOESM1_ESM.tif]

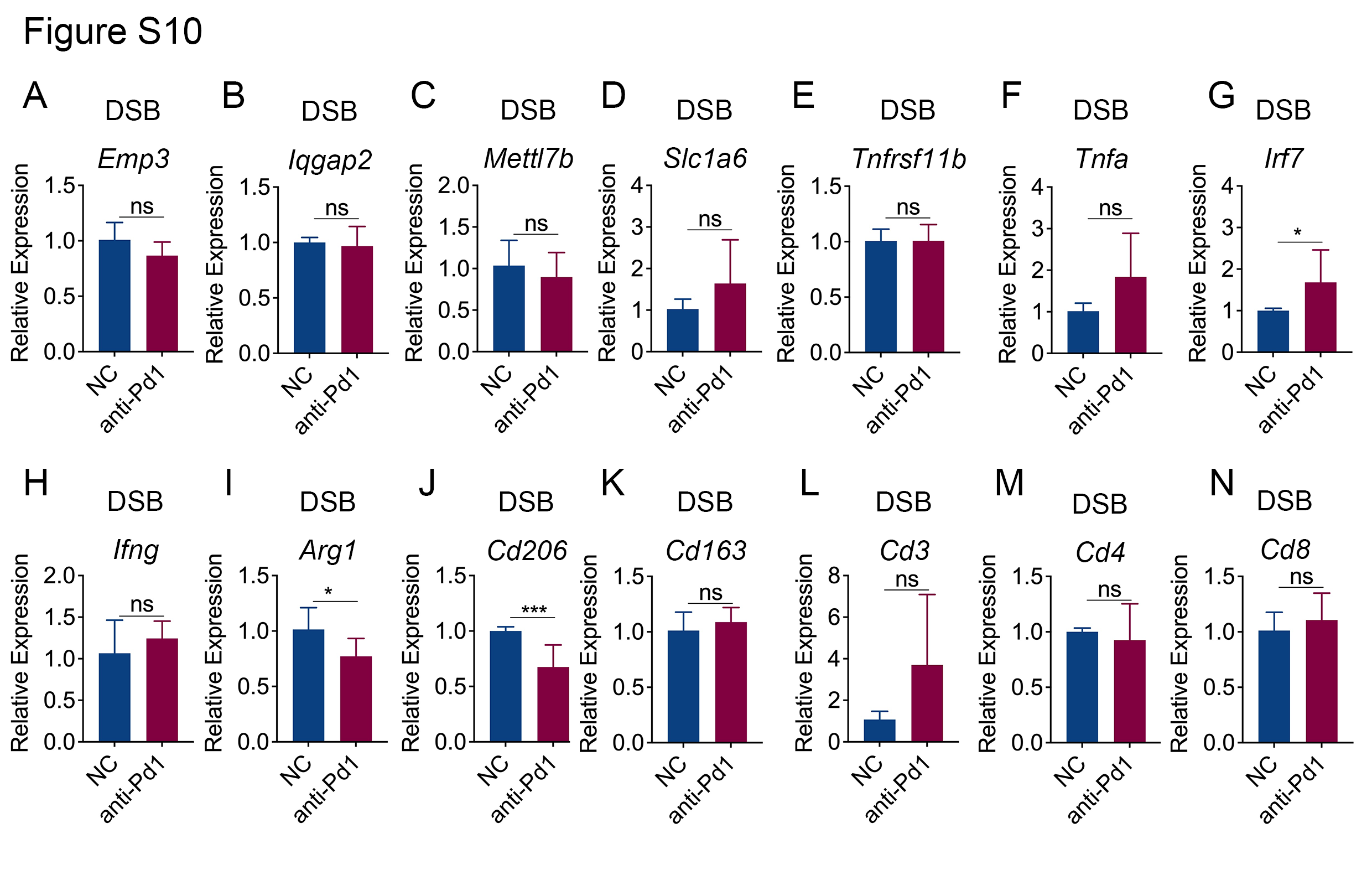

Supplement: Supplementary file 2 — Additional file 2: Fig. S2. Four immune subtypes with different immune cell infiltration status in LGG. [file 12885_2023_11222_MOESM2_ESM.tif]

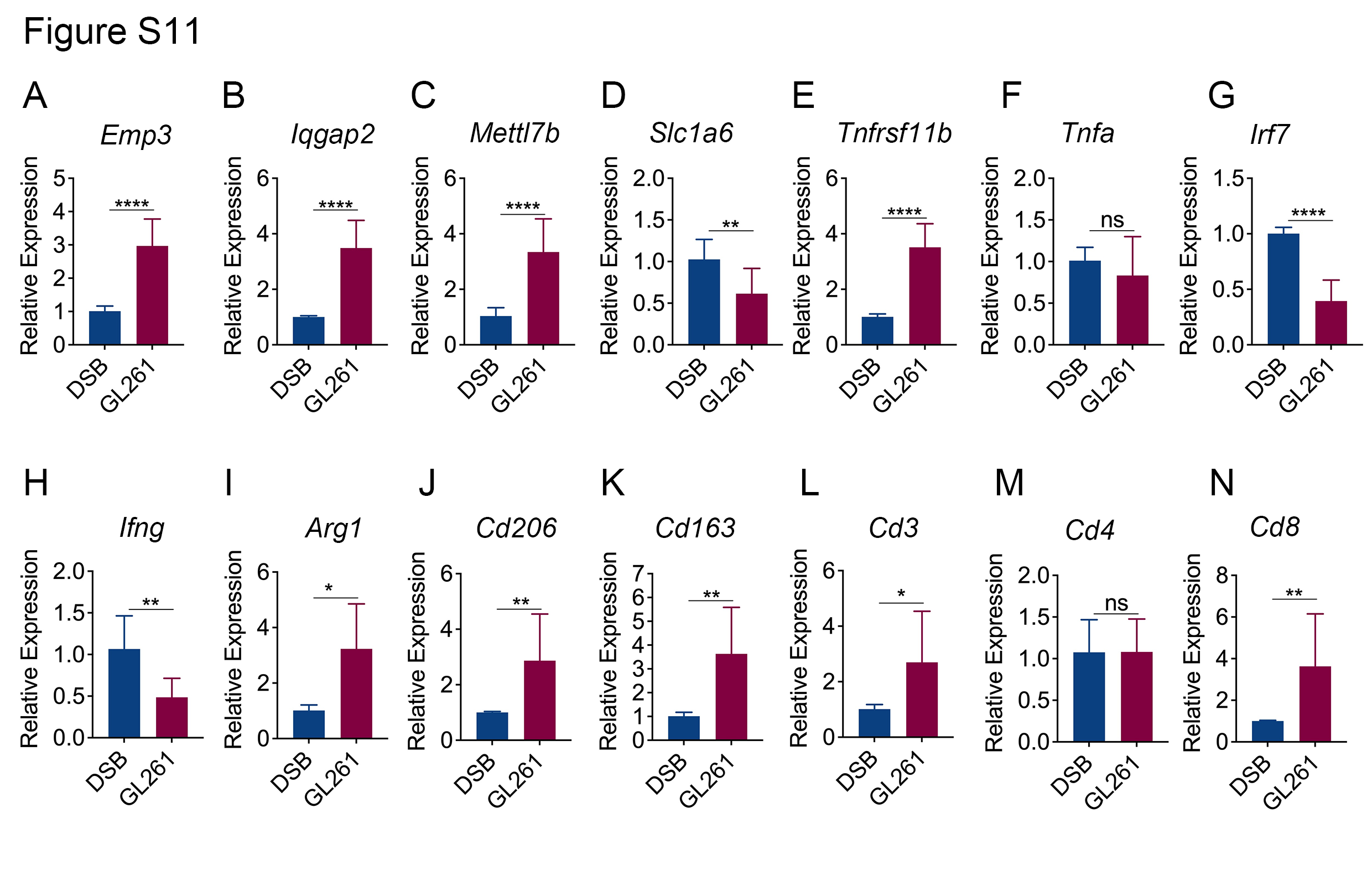

Supplement: Supplementary file 3 — Additional file 3: Fig. S3. Signal pathways and biological processes enriched in the low immune infiltration subtype. [file 12885_2023_11222_MOESM3_ESM.tif]

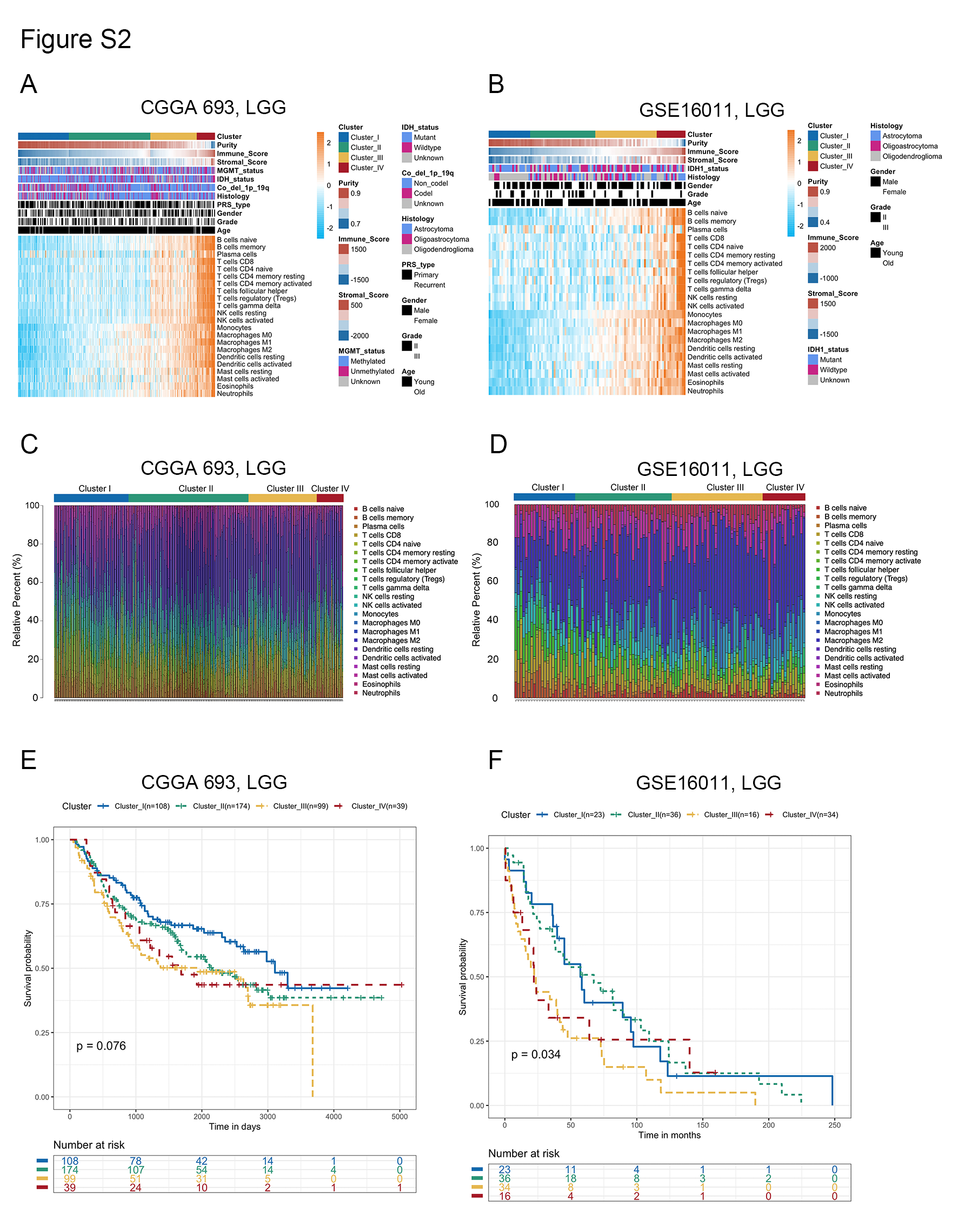

Supplement: Supplementary file 4 — Additional file 4: Fig. S4. Construction and validation of IIS in LGG. [file 12885_2023_11222_MOESM4_ESM.tif]

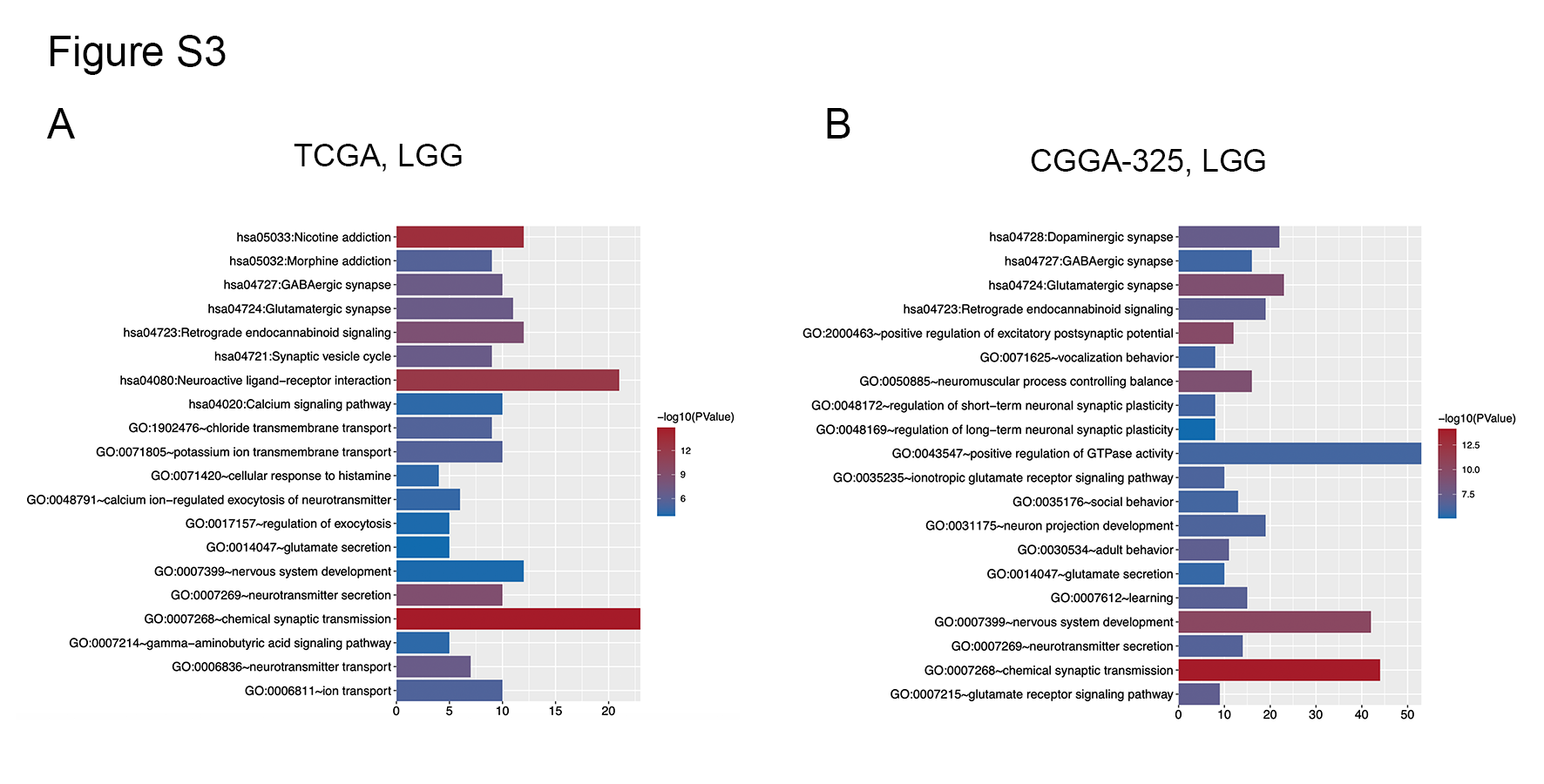

Supplement: Supplementary file 5 — Additional file 5: Fig. S5. Establishment and validation of IIS risk score nomogram. [file 12885_2023_11222_MOESM5_ESM.tif]

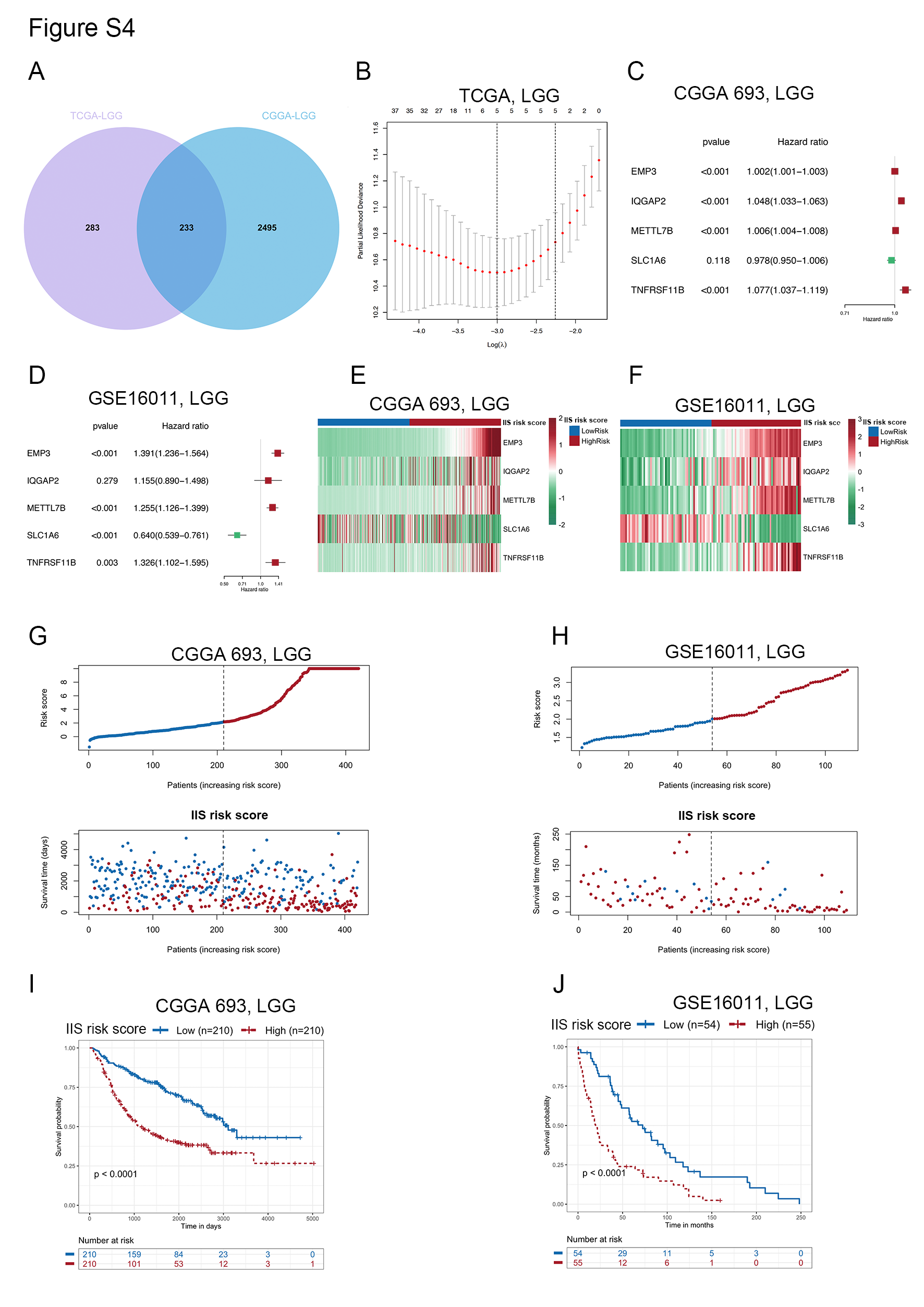

Supplement: Supplementary file 6 — Additional file 6: Fig. S6. Correlation between IIS risk score and clinicopathological features. [file 12885_2023_11222_MOESM6_ESM.tif]

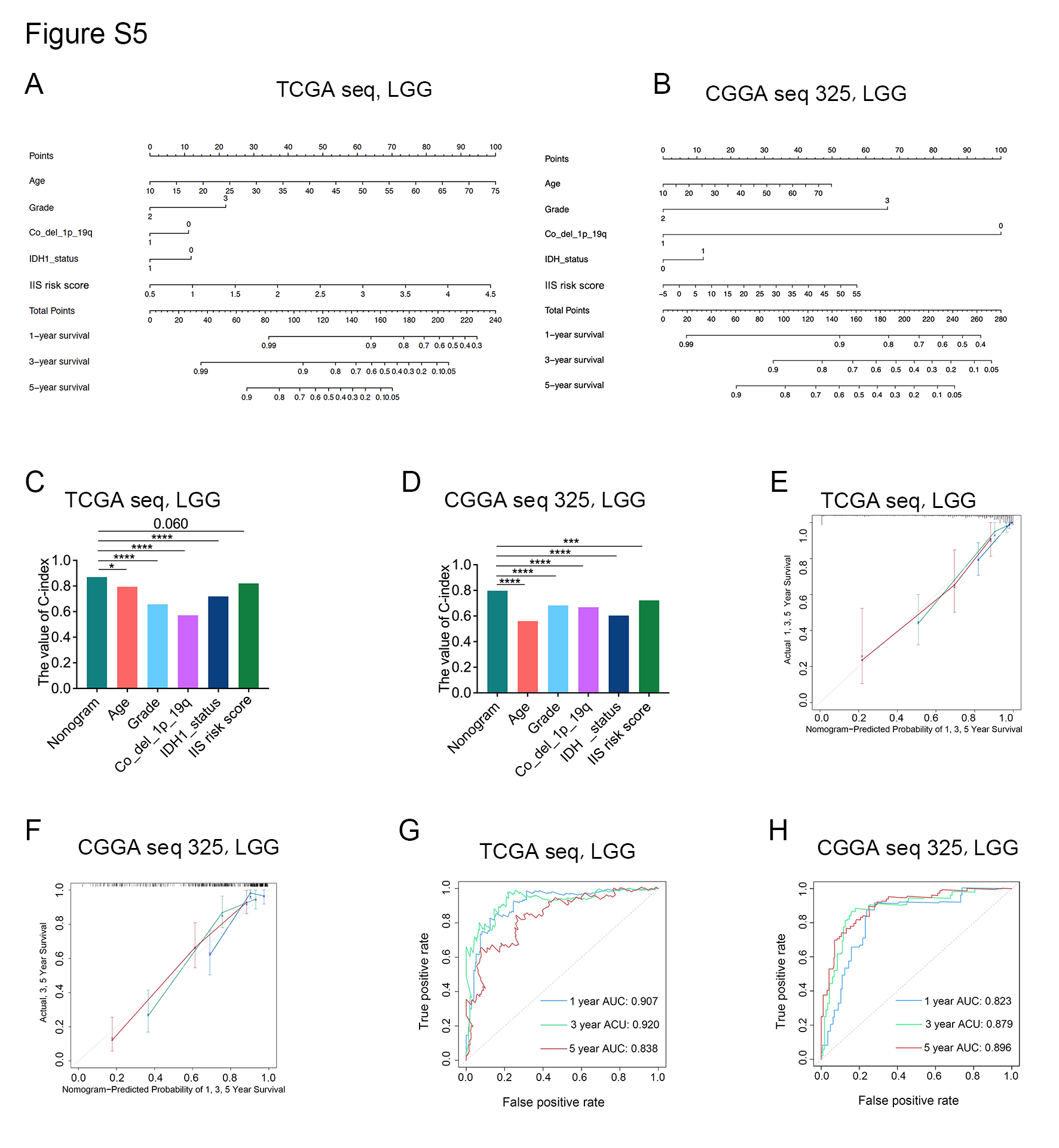

Supplement: Supplementary file 7 — Additional file 7: Fig. S7. Correlation of IIS risk score with non-tumor immune populations in the TME. [file 12885_2023_11222_MOESM7_ESM.tif]

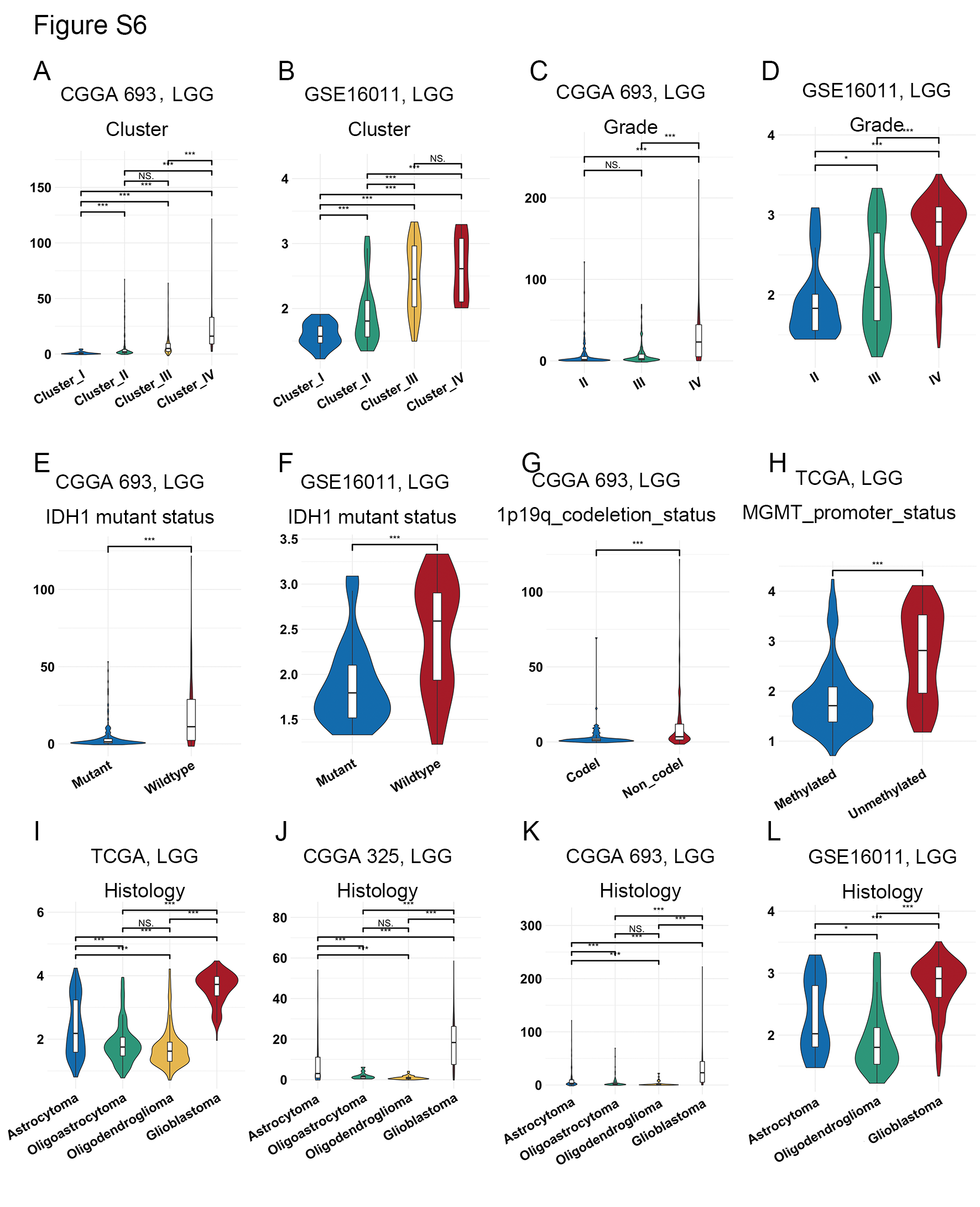

Supplement: Supplementary file 8 — Additional file 8: Fig. S8. LGG with different IIS risk score had distinct genomic and transcriptomic spectrum. [file 12885_2023_11222_MOESM8_ESM.tif]

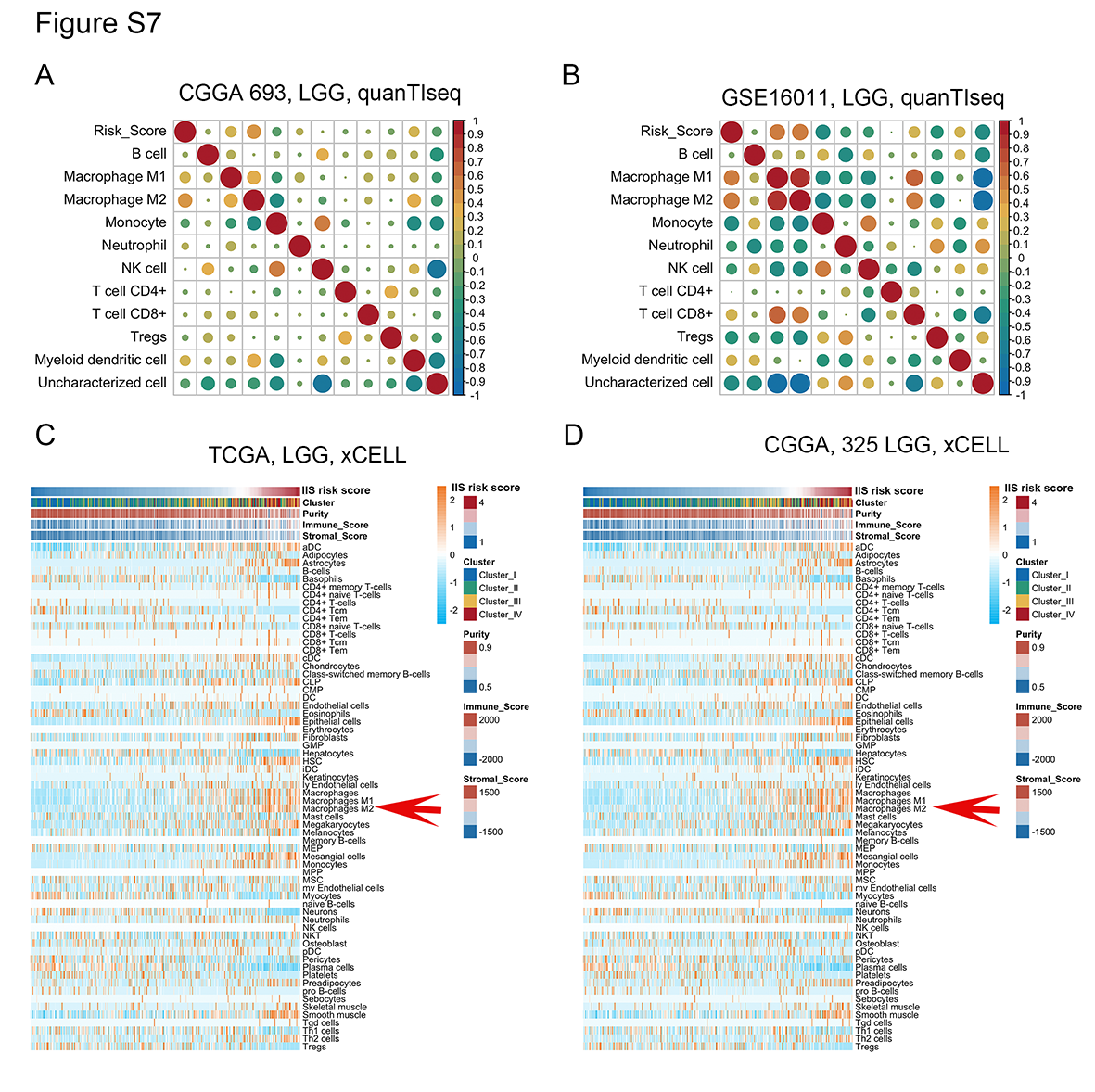

Supplement: Supplementary file 9 — Additional file 9: Fig. S9. IIS risk score was associated with immunomodulatory molecules and could predict response of immunotherapy. [file 12885_2023_11222_MOESM9_ESM.tif]

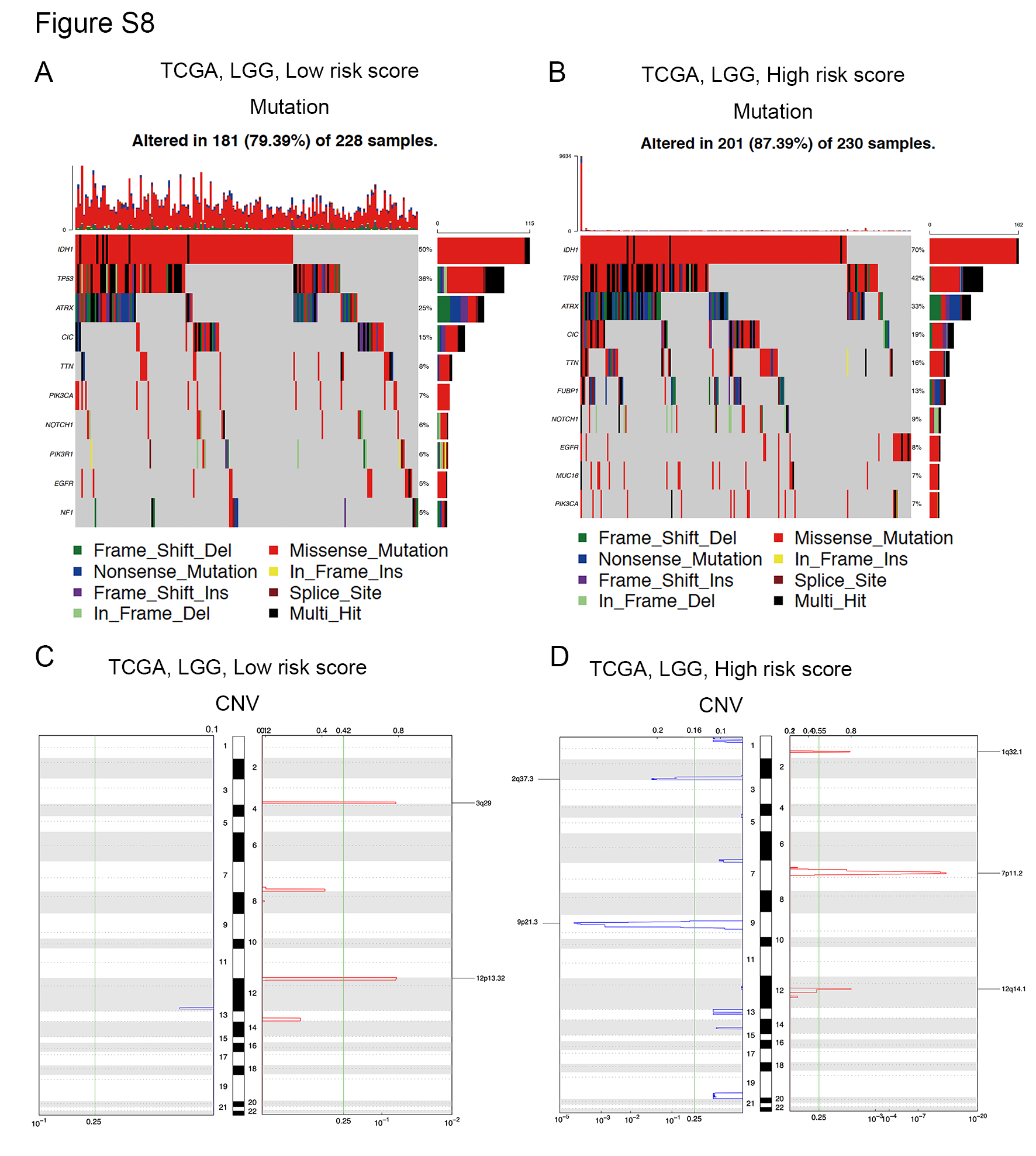

Supplement: Supplementary file 10 — Additional file 10: Fig. S10. There were no significant changes in IIS risk score and immune cell infiltration in the immunotherapy unresponsive group. [file 12885_2023_11222_MOESM10_ESM.tif]

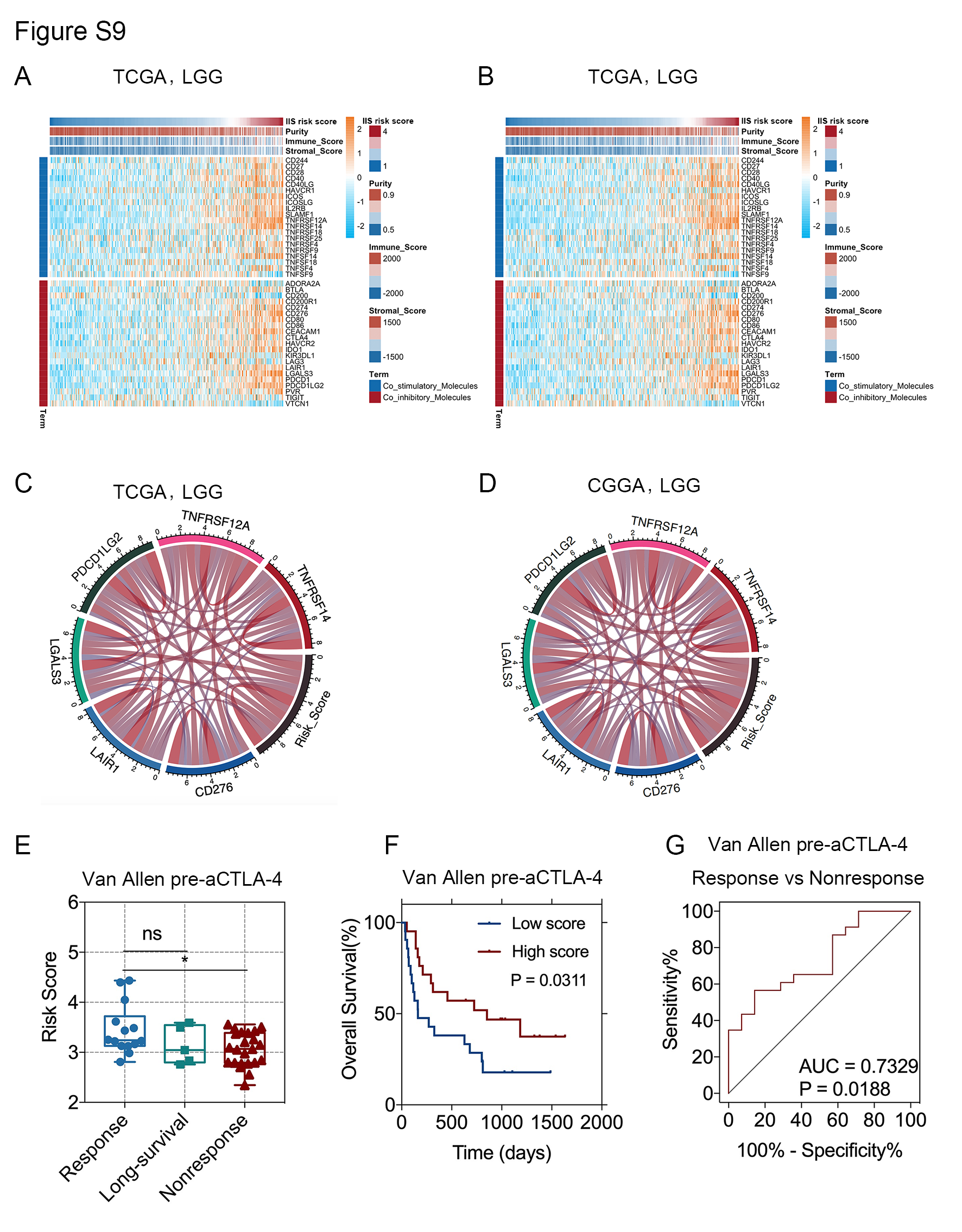

Supplement: Supplementary file 11 — Additional file 11: Fig. S11. The IIS risk score could predict the degree of immune cell infiltration and the response to immunotherapy in glioma. [file 12885_2023_11222_MOESM11_ESM.tif]
